# Emergent bound states and impurity pairs in chemically doped Shastry-Sutherland system

**DOI:** 10.1038/s41467-019-10410-x

**Published:** 2019-06-04

**Authors:** Zhenzhong Shi, William Steinhardt, David Graf, Philippe Corboz, Franziska Weickert, Neil Harrison, Marcelo Jaime, Casey Marjerrison, Hanna A. Dabkowska, Frédéric Mila, Sara Haravifard

**Affiliations:** 1 Department of Physics, Duke University, Durham, NC 27708 USA; 2 National High Magnetic Field Laboratory, Florida State University, Tallahassee, FL 32310 USA; 3 Institute for Theoretical Physics and Delta Institute for Theoretical Physics, University of Amsterdam, Science Park 904, 1098 XH Amsterdam, The Netherlands; 4 National High Magnetic Field Laboratory, Los Alamos National Laboratory, Los Alamos, NM 87545 USA; 5 Brockhouse Institute for Material Research, McMaster University, Hamilton, Ontario, L8S 4M1 Canada; 6 Institute of Physics, École Polytechnique Fédérale de Lausanne (EPFL), CH-1015 Lausanne, Switzerland; 7 Department of Mechanical Engineering and Materials Science, Duke University, Durham, NC 27708 USA

**Keywords:** Magnetic properties and materials, Phase transitions and critical phenomena

## Abstract

Impurities often play a defining role in the ground states of frustrated quantum magnets. Studies of their effects are crucial in understanding of the phase diagram in these materials. SrCu_2_(BO_3_)_2_, an experimental realization of the Shastry-Sutherland (SS) lattice, provides a unique model system for such studies using both experimental and numerical approaches. Here we report effects of impurities on the crystals of bound states, and doping-induced emergent ground states in Mg-doped SrCu_2_(BO_3_)_2_, which remain stable in high magnetic fields. Using four complementary magnetometry techniques and theoretical simulations, a rich impurity-induced phenomenology at high fields is discovered. The results demonstrate a rare example in which even a small doping concentration interacts strongly with both triplets and bound states of triplets, and thus plays a significant role in the magnetization process even at high magnetic fields. Our findings provide insights into the study of impurity effects in geometrically frustrated quantum magnets.

## Abstract (web summary)

Exploring the impurity-induced phenomena facilitates the understanding of emergent quantum materials. Here the authors show the anomalous magnetization transitions as well as demonstrate the relation between the impurities and the excited spin states in the Mg doped Shastry-Sutherland compound SrCu_2_(BO_3_)_2_.

## Introduction

Geometrical frustration in low-dimensional quantum spin systems underlies many of the exotic states of matter that are of great current interest in condensed matter physics^1^. One of the central topics in studying such systems is to understand the effects of impurities that are unavoidable in realistic materials^2–5^ and sometimes intentionally introduced by chemical doping^6^. However, such studies are often hindered by the complex Hamiltonians in analytical and numerical studies, and difficulties in finding a suitable model system to make a direct comparison between theoretical calculations and experimental results. Here we show that SrCu_2_(BO_3_)_2_, a realization of the exactly solvable SS model^7,8^, provides an important test ground for our understanding of the effects of impurities in frustrated quantum magnets.

**Fig. 1:**
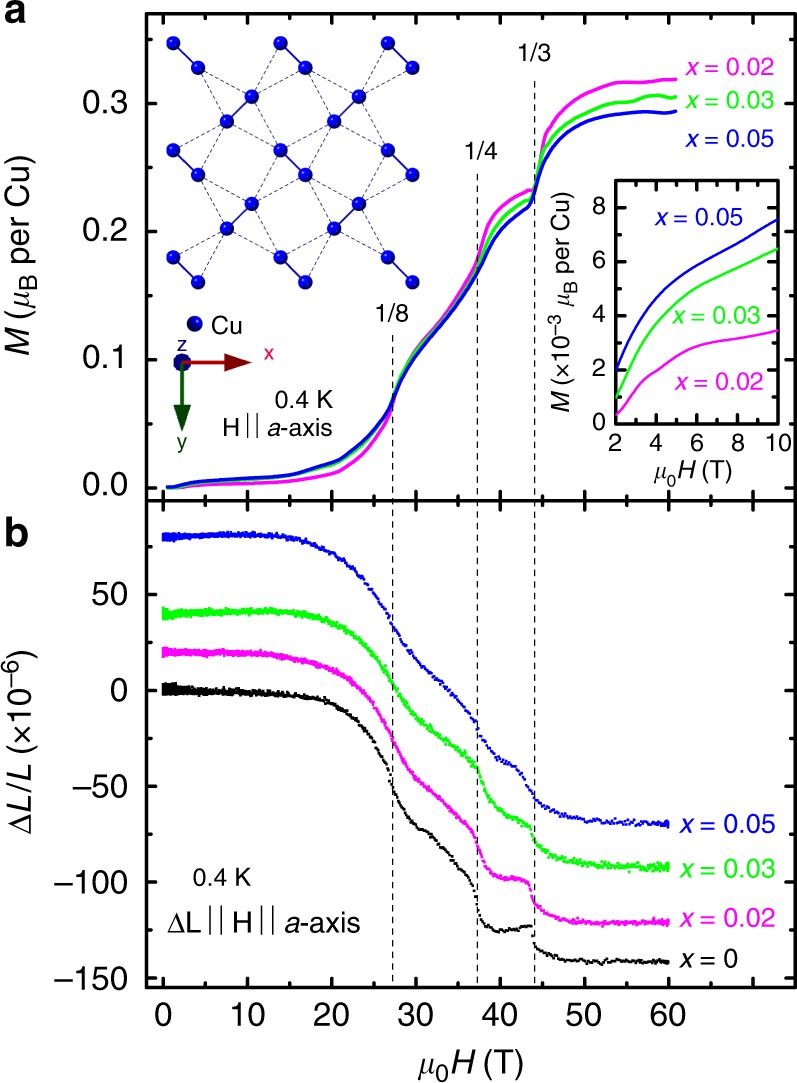
Magnetization plateaus in Mg-doped SrCu_2_(BO_3_)_2_. **a** Magnetization (*M*) vs. field (**H** ∥ *a*-axis) for *x* = 0.02 (magenta), 0.03 (green), and 0.05 (blue), and **b** magnetostriction (∆*L*/*L*) vs. field (**H** ∥ *a*-axis) for *x* = 0 (black), 0.02 (magenta), 0.03 (green), and 0.05 (blue), at *T* = 0.4 K, conducted in a 65 T multi-shot magnet at the pulsed field facility of the national high magnetic field laboratory (NHMFL). Lower right inset: *M*(*H*) for the three dopings at low *H*, obtained from 60 T shots. Upper left inset: a schematic of the spin-1/2 Cu^2+^ ions in the SS lattice, as realized in SrCu_2_(BO_3_)_2_. Traces in (**b**) are shifted for clarity

The parent compound of SrCu_2_(BO_3_)_2_ consists of two-dimensional layers of Cu^2+^(*S* *=* 1/2) orthogonal dimers arranged on a square lattice (Fig. 1a inset), which form the *ab*-plane of its tetragonal unit cell. A spin gap ∆ ∼ 3 meV separates the *S* *=* 0 singlet ground state from an *S* *=* 1 triplet excited state. Without doping, SrCu_2_(BO_3_)_2_ has a ground state of a valence bond solid at low temperature and zero magnetic field. Studies on doped SrCu_2_(BO_3_)_2_ have been largely motivated by the prospect of resonating valence bond (RVB) superconductivity, when holes are doped into this material^8–12^, and the effects of the doping-introduced *S* *=* 1/2 spin singlet impurities on its ground state remain to be thoroughly explored.

To understand the ground state induced by the interplay among the non-magnetic spin singlets, the magnetic spin triplet excitations, and the doping-introduced *S* = 1/2 spin singlet impurities at low energies, we have studied SrCu_2−*x*_Mg_*x*_(BO_3_)_2_, in which the magnetic Cu^2+^ is substituted with non-magnetic isoelectronic Mg^2+^, introducing minimal structure distortion because of their similar ionic radii. Previous inelastic neutron scattering and *µ*SR experiments on SrCu_2−*x*_Mg_*x*_(BO_3_)_2_(*x* = 0.05) have shown that some dimers are indeed broken, and in-gap states emerge^13,14^. It was suggested that the in-gap states might correspond to localized anisotropic spin polarons developed around the impurities^13,15^, or to the *S* = 1/2 states that consist of one spinon and one impurity^16^. However, a clear understanding for the effects of non-magnetic impurities in SS systems remains elusive. Here we demonstrate that critical insights are gained by studying the magnetization response of the Mg-doped SrCu_2_(BO_3_)_2_ in high magnetic fields.

In the presence of magnetic field, frustration has been known to induce magnetization plateaus, occurring at fractional values of saturation magnetization *M*_sat_, either due to a classical mechanism involving stabilization of some classical spin configurations, or due to a quantum mechanism which corresponds to symmetry-breaking phase transitions in an effective hard-core-boson model^1^. As one of the best examples of the latter case, SrCu_2_(BO_3_)_2_ exhibits a series of magnetization plateaus at magnetic fields above which the spin gap is closed by the Zeeman energy^17–22^. This has been understood as a result of the crystallization of *S*_*z*_ = 2 pinwheels of bound states of two triplets^23^, which are energetically more favorable than crystals of *S*_*z* _ = 1 triplets. This picture is well established in the undoped system. In the doped system, however, it is not clear how the added impurities would interact with the triplets and bound states of triplets, and hence alter their crystallization.

Here, we report a comprehensive doping dependence study of the magnetometry in high magnetic fields, revealing a surprisingly rich impurity-induced phenomenology in these systems: doping-induced triplet states and emergent impurity pairs. It was found that the conventional magnetization measurements alone do not provide a full picture explaining the subtle changes associated with such a study. Therefore, we combined four complementary techniques: tunnel diode oscillator (TDO) and torque magnetometry, which measure magnetic susceptibility; magnetization measurements, which probe magnetization response directly; magnetostriction measurements, which detect lattice correlations to the magnetic order in very high magnetic fields. The results are in remarkable agreement with our numerical simulations using infinite projected entangled pair states (iPEPS), providing an accurate account of the various impurity-induced emergent states. Our results offer essential implications for the understanding of doped quantum spin systems.

## Results

### Magnetization plateaus

The TDO and torque magnetometry experiments were conducted at the National High Magnetic Field Laboratory (NHMFL) dc field facility, while the magnetization and magnetostriction measurements were carried out at the NHMFL pulsed field facility. Single crystals of SrCu_2_(BO_3_)_2_ and SrCu_2−*x*_Mg_*x*_(BO_3_)_2_, with *x* up to 0.05, were grown using the optical floating zone technique (see “Methods”). In the doped samples, the magnetic Cu^2+^ sites are replaced with non-magnetic isoelectronic Mg^2+^, which effectively breaks the spin dimers into free *S* = 1/2 spins, without introducing structural distortions. The doping concentrations were confirmed by susceptibility measurements (see Supplementary Fig. 1).

We show in Fig. 1a the magnetization response for SrCu_2−*x*_Mg_*x*_(BO_3_)_2_ (*x* = 0.02, 0.03, and 0.05) at 0.4 K with **H** ∥ *a*-axis up to 60 T. At low fields, a notable finite magnetization, which increases with doping, is observed (see Fig. 1a lower right inset). For *H* smaller than 6 ∼ 8 T, *M*(*H*) exhibits a Brillouin-like paramagnetic behavior for all three dopings, and the results are consistent with the field-induced alignment of free *S* = 1/2 impurity spins. However, a full saturation of magnetization is interrupted at *H* above 6 ∼ 8 T, suggesting a more complicated picture than one solely explained by the impurity-induced free spins; as will be discussed later. The sharp onset of magnetization at *H* higher than ∼18 T is attributed to the increase in population of triplets, as spin gap closes with increasing field^1^. In doped SrCu_2_(BO_3_)_2_, Fig. 1a clearly shows that the magnetization is suppressed with increasing doping, suggesting a suppressed density of triplets in the presence of impurities. Nevertheless, for all doping concentrations, magnetization plateaus similar to those in the undoped case are observed at the same onset fields, albeit with important differences as discussed below.

**Fig. 2:**
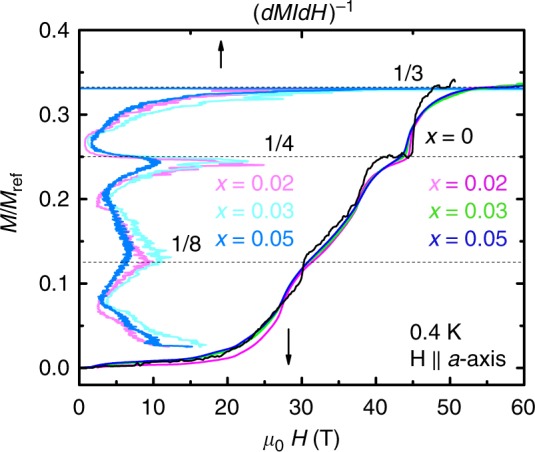
Magnetization normalized by the reference magnetization *M*_ref_. M/M_ref_ vs. *µ*_0_*H* (bottom axis) for *x* *=* 0 (black), 0.02 (magenta), 0.03 (green), and 0.05 (blue), with **H** ∥ *a*-axis, at *T* = 0.4 K. Plateaus are indicated by peaks in the inverse susceptibility (*dM*/*dH*)^−1^ (top axis) for *x* = 0.02 (light magenta), 0.03 (light cyan), and 0.05 (light blue). Dashed lines guide the eye. The magnetization values at the 1/3 (pseudo-1/*n*) plateau are used to extract the reference saturation magnetization, *M*_ref _ = 1.065, 0.952, 0.913, and 0.881 *µ*_B_ per Cu for the *x* = 0, *x* = 0.02, *x* = 0.03, and *x* = 0.05 samples, respectively. Here, the *x* = 0 trace (black) of *M* vs. *µ*_0_*H* is reproduced from a **H** ∥ *c*-axis trace (adapted with permission from, ref. ^24^. copyright American Physical Society 2005, also see Supplementary Note 1), rescaled to allow comparison with our **H** ∥ *a*-axis data on the doped samples

First, the plateaus in the doped systems can no longer be identified as fractions (1/8, 1/4, 1/3, …) of the saturation magnetization *M*_sat_, where all the magnetic Cu^2+^ moments are fully saturated. Therefore, for clarity, these plateaus are named as pseudo-1/8, pseudo-1/4, and pseudo-1/3 plateaus in the doped systems, to be differentiated from the 1/8, 1/4, and 1/3 plateaus in the undoped case. Second, a term that is more general than *M*_sat_ can be defined: the reference magnetization *M*_ref_. In the undoped system, *M*_ref_ is the same as *M*_sat_, and the magnetization plateaus occur at fractions (1/8, 1/4, 1/3, …) of *M*_sat_ (=*M*_ref_). In the doped system, however, the magnetization values of the pseudo-1/*n* (*n* = 2, 3, 8,…) plateaus are found to be fractions (1/8, 1/4, 1/3, …) of a doping-dependent magnetization value, which is different from the true saturation magnetization *M*_sat_ of these doped systems, as demonstrated later. This magnetization value is identified as *M*_ref_. It can be extracted from the pseudo-1/3 plateaus for the doped system (or 1/3 plateau for the undoped case): *M*_ref_ = 3 × *M*_1/3_, where *M*_1/3_ refers to the magnetization at the pseudo-1/3 (or 1/3) plateau. We plot in Fig. 2 the normalized magnetization curves, *M*/*M*_ref_, as function of field, for both the undoped and doped samples. Here, it is easy to verify that the pseudo-1/8 and pseudo-1/4 plateaus in the doped systems have magnetization values that are approximately 1/8 and 1/4 of *M*_ref_, similar to the undoped case. Using this method, *M*_ref_ are found to be 1.065, 0.952, 0.913, and 0.881 *µ*_B_ per Cu for the *x* = 0, 0.02, 0.03, and 0.05 samples, respectively (see Figs. 1a and 2). Note that the *x* = 0 data is reproduced from ref. ^24^ (see Supplementary Note 1).

The observation that the values of *M*_ref_ decrease upon doping is rather interesting. One might expect that in the doped systems, *M*_1/3_ at the pseudo-1/3 plateau has contributions from both superstructures of bound states^23^ (as in the undoped samples) and the free spins located on broken dimers with impurity sites (up to 2.5% with the highest doping *x* = 0.05), and therefore, the value of *M*_ref_ (3*M*_1/3_) would be slightly larger than the saturation magnetization, due to the overvaluation of the magnetization of the free spins. This is obviously not the case.

Moreover, as mentioned above, *M*_ref_ is also different from the true saturation magnetization *M*_sat_ in the doped systems. For example, *M*_sat_ for the *x* = 0.05 sample is expected to be 2.5% smaller than that in the undoped case, because of the loss of 2.5% of magnetic Cu^2+^ moments. However, *M*_ref_ for the same doping (0.881 *μ*_*B*_ per Cu) is found to be ∼17% less than *M*_sat_ (=*M*_ref_) for the undoped sample (1.065 *μ*_*B*_ per Cu). Therefore, *M*_ref_ must reflect an intrinsic change in the spin superstructures as a result of doping. Indeed, it is not surprising that doping with nonmagnetic static impurities would disrupt and soften the superstructure of the bound states underlying the magnetization plateaus: the formation of the 1/3 superstructure is perturbed in a certain neighborhood of the impurities, resulting in patches of superstructures rather than a perfect 1/3 superstructure with 2.5% of the sites removed. In this scenario, *M*_ref_ is the total magnetization of all the patches of superstructures and does not include the nonmagnetic spin singlets which lie in between. The fact that *M*/*M*_ref_ curves for the undoped^24^ and doped samples overlap and show the same sequence for plateaus at 1/8 (pseudo-1/8), 1/4 (pseudo-1/4), and 1/3 (pseudo-1/3), as seen in Fig. 2, suggests that the undoped and doped systems share the same underlying superstructures^23^ at these plateaus, enabled by some collaborative geometrical arrangement of the impurities and the triplets in the doped samples. The same onset fields of these (pseudo-1/*n*) plateaus also suggest the excitation energy (or effective chemical potential) of the superstructures of the bound states of triplets does not depend on doping. The smearing of the pseudo-1/*n* plateaus with doping is consistent with the softening of the superstructures in the system. For instance, the volume of the superstructures in the undoped system is close to 100% of the total volume of the sample, and it is reduced to *M*_ref_/*M*_sat_ ~ 85% for the *x* = 0.05 sample. At the highest fields, however, all the moments are fully saturated, and the magnetization should eventually reach *M*_sat_. It is interesting to speculate how this process takes place, and extremely high magnetic fields are required for such a study.

Complementary magnetostriction measurements (see “Methods”) performed for both the undoped and doped samples are plotted in Fig. 1b and Supplementary Fig. 2. These results reveal contraction along the *a*-axis, which closely corresponds to changes in magnetization and are consistent with previous results reported for the undoped system^20,25^. Furthermore, these results clearly show the (pseudo-) 1/8, (pseudo-) 1/4, and (pseudo-) 1/3 plateaus, for which the onset fields agree very well with those determined from the magnetization measurements. The increasingly softened pseudo-1/*n* plateaus with doping also suggest that the overall lattice coupling is suppressed with the increased density of impurities.

### Emergent magnetization states at low *H*

**Fig. 3:**
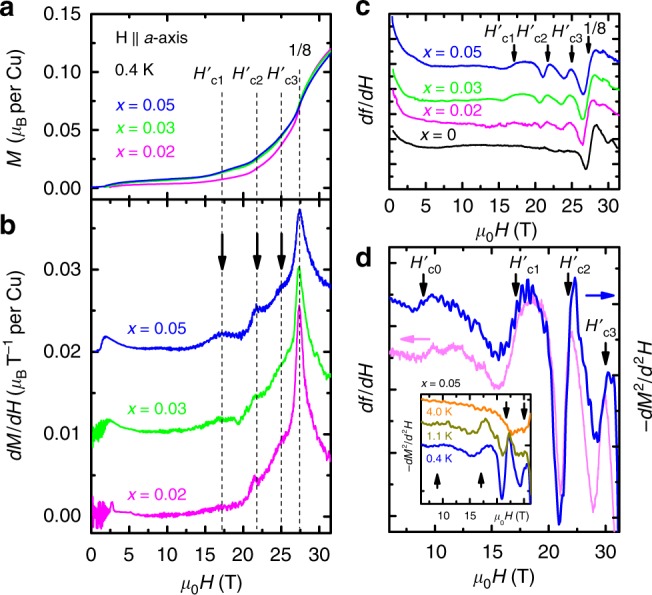
Emergence of magnetization anomalies below the 1/8 plateau. Field dependence of **a**, *M* and **b**, *dM*/*dH*, for three doping levels *x* = 0.02 (magenta), 0.03 (green), and 0.05 (blue), with **H** ∥ *a*-axis, at *T* = 0.4 K. The feature corresponding to the pseudo-1/8 plateau is marked by a dashed line at ~27 T. *H*’_C1_, *H*’_C2_, and *H*’_C3_ and the corresponding dashed lines indicate three of the additional anomalies below the pseudo-1/8 plateau. For clarity, a shift of 0.01 (0.02) *µ*_B_*T*^-1^ per Cu is added to the *x* = 0.03 (0.05) trace. **c** The first derivative of the frequency shift, *df*/*dH* (∝ *dM*^2^/*d*^2^*H*), as a function of applied field for *x* =   0 (black), 0.02 (magenta), 0.03 (green), and 0.05 (blue), measured by the tunnel diode oscillator technique (see “Methods”) at the dc field facility of NHMFL. Upon doping, three anomalies emerge at *H*’_C1_, *H*’_C2_, and *H*’_C3_, consistent with the magnetization measurements in (**b**). **d** A zoomed-in plot of *df*/*dH* (light magenta, left axis) and −*dM*^2^/*d*^2^*H* (blue, right axis) vs. *µ*_0_*H* for the *x* = 0.05 sample, shows an additional broad anomaly at a lower field *H*’_C0_. Inset: −*dM*^2^/*d*^2^*H* vs. *µ*_0_*H* for a few selective temperatures. The arrows, from left to right, indicate *H*’_C0_, *H*’_C1_, *H*’_C2_, and *H*’_C3_, respectively. Traces in (**c**) and inset of (**d**) are shifted for clarity

**Fig. 4:**
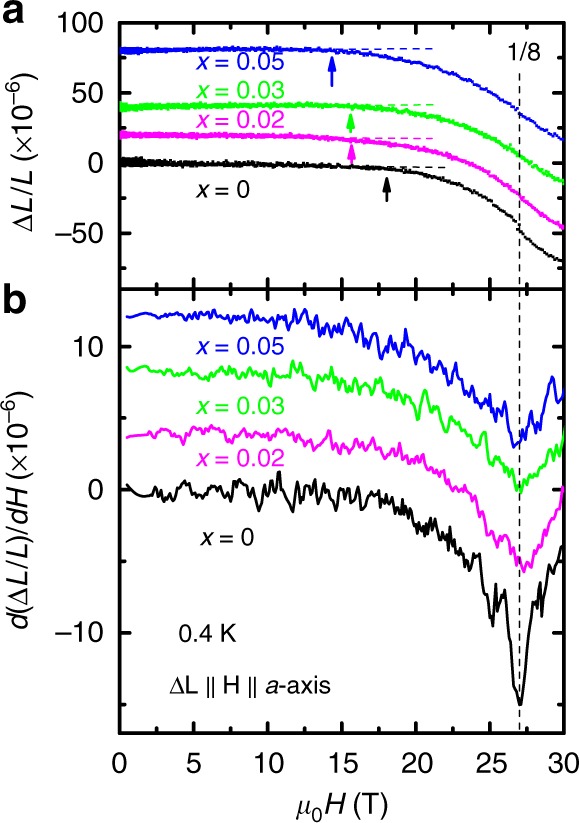
Absence of the magnetization anomalies in magnetostriction measurements. Field dependence of **a**, relative deformation ∆*L*/*L* along the tetragonal axis, and **b**, its first derivative *d*(∆*L*/*L*)/*dH*, with ∆**L** ∥ **H** ∥ *a*-axis, at *T* *=* 0.4 K, for *x* = 0 (black), 0.02 (magenta), 0.03 (green), and 0.05 (blue). The arrows in **a** point to the onsets of deviation from linear fits to the low-field regions for each doping (dashed lines). The vertical dashed line marks the (pseudo-) 1/8 plateau, identified as the local minima in *d*(∆*L*/*L*)/*dH* vs. *µ*_0_*H*. Traces are shifted for clarity

Our most important results are obtained from a close examination of the magnetization response for the doped samples in the field region below the pseudo-1/8 plateau, as presented in Figs. 3 and 4. In fact this region is of broad interest, though not well understood even in the undoped system. For example, other than the 1/9 plateau^19,20^, spin superstructures with even smaller fractions, i.e., larger unit cells, remain elusive. Theoretical considerations seem to suggest that they are energetically favorable only in very limited field ranges, if at all possible^23^. In a doped system, the phase diagram becomes even richer as the density of impurities increases.

We plot in Fig. 3a and b the magnetization curves as function of *x* for the low-field region. Indeed, the magnetization measurements clearly show three low-field anomalies, i.e., jumps in *M*(*H*) curves, as can be seen for all doped samples. For the *x* = 0.05 sample, the onset fields of these anomalies are determined as *H*’_C1_ ∼ 17.1 T, *H*’_C2_ ∼ 21.7 T, and *H*’_C3_ ∼ 25.0 T (see Supplementary Figs. 3a and 4). For the *x* = 0.02 and 0.03 samples, three anomalies are also identified at similar fields. Figure 3b shows the magnitudes of these anomalies, measured by *dM*/*dH*, are much smaller than that of the pseudo-1/8 plateau for all *x*. Their doping dependence, however, are exactly the opposite: the *H*’_C1_, *H*’_C2_, and *H*’_C3_ anomalies are enhanced with higher doping concentration, while the pseudo-1/8 plateau is suppressed. This completely different behavior on doping suggests that these anomalies have origins that are different from the pseudo-1/*n* plateaus. The broad maxima at very low *H* ∼ 1 T − 2 T are attributed to field-aligned free *S* = 1/2 impurity spins, associated with the onset of finite magnetization, as discussed above.

We plot in Fig. 3c and d the results for TDO magnetic susceptibility measurements (see Methods), where *df*/*dH* is proportional to *dM*^2^/*d*^2^*H*; the corresponding comparison is clearly shown in Fig. 3d for the *x* = 0.05 sample. The TDO measurements performed in a quieter magnet environment, i.e., a steady magnetic field instead of a pulsed field, show more clearly the emergence of the *H*’_C1_, *H*’_C2_, and *H*’_C3_ anomalies with doping, and their absence in the undoped system (see Fig. 3c). This is consistent with the fact that these anomalies were never observed in the undoped system^19,21,24^. Strikingly, another broad anomaly at *H*’_C0_ ∼ 9 T, which is much weaker than its higher field counterparts, is only observed for the highest doping concentration *x* = 0.05 sample. The confirmation of such a weak anomaly underlines the importance of adopting different techniques for measuring the same physical quantity when the signal is weak. These anomalies are found to be suppressed with increasing temperature (see Fig. 3d inset, Supplementary Figs. 5 and 6). In the *x* = 0.05 samples, for example, they disappear at *T* ∼ 2 K, before the pseudo-1/*n* plateaus melt at ~3 K−4 K (see Supplementary Figs. 2d and 5d). The different energy scales suggest that the underlying spin structures of these anomalies are different from the superstructures of bound states of triplets associated with the pseudo-1/*n* plateaus. This is confirmed by our simulation results as discussed below.

The coupling of these anomalies to the lattice is investigated using magnetostriction measurements. In both undoped and doped samples, the axial magnetostriction along *a*-axis deviates from zero at fields that gradually decrease from ∼18 T in *x* = 0 to ∼14 T in the *x* = 0.05 sample, as indicated by arrows in Fig. 4a. As can be seen in Fig. 4b, however, no anomalies are observed for any of the samples at fields below the pseudo-1/8 plateau. Though a lack of sufficient resolution cannot be completely ruled out, the absence of these anomalies in the magnetostriction data suggests their weak coupling to lattice. This is unlike the strong lattice coupling observed for magnetization plateaus corresponding to the crystallization of bound states of triplets, confirming their different origins. This interpretation is further strengthened by the fact that the *H*’_C1_ and *H*’_C0_ anomalies appear at fields comparable with, or below, the gap closing fields at which bound states of triplets are absent and cannot play any role in their formation. Indeed, iPEPS numerical simulations clearly demonstrate that the observed anomalies all have impurity-induced origins, as we explain in the following sections.

### Infinite projected entangled pair states

Our simulation results are obtained using iPEPS—a variational tensor network ansatz to represent a 2D ground state directly in the thermodynamic limit^26–28^. The ansatz consists of a unit cell of tensors which is periodically repeated on the infinite lattice, where in the present case we use one tensor per dimer^23,29,30^. The accuracy of the ansatz can be systematically controlled by the bond dimension *D* of the tensors.

The optimization of the variational parameters has been done using the simple update method which provides good estimates of ground state energies while being computationally affordable, even in the limit of very large unit cell sizes (up to 12 × 12 dimers in the present work). For the computation of observables, a variant^31,32^ of the corner-transfer matrix method^33,34^ is used. To improve the efficiency, we exploit the U(1) symmetry of the model^35,36^. For an introduction to the method, see refs. ^37,38^ for example.

### Model used for the Mg-doped SrCu_2_(BO_3_)_2_

A well-established effective model to describe the low-energy physics of SrCu_2_(BO_3_)_2_ is the SS model^2^ given by the Hamiltonian

1 $$H = J\mathop {\sum }\limits_{(i,j)} S_i \cdot S_j + J\prime \mathop {\sum }\limits_{(i,j)} S_i \cdot S_j - h\mathop {\sum }\limits_i S_i^z$$

where the bonds with coupling strength *J* build an array of orthogonal dimers and the bonds with coupling *J*′ denote inter-dimer couplings, and *h* is the strength of the external magnetic field. In the present work we use *J*′/*J* *=* 0.63 (with *J* ∼ 51 T) which was obtained from a fit to high magnetic field data^30^.

At zero external magnetic field, the ground state is given by a product of singlets on the dimers^2^. Early on, it was found that the SS model has almost localized triplet excitations^39,40^ which has led to the viewpoint that the magnetization plateaus found in SrCu_2_(BO_3_)_2_ correspond to crystals of triplets^39,41–51^. However, it was predicted that *S*_*z*  _= 2 excitations, which can be seen as a bound state of two triplets, are energetically lower in the dilute limit of excitations^42^. Based on iPEPS simulations, it was shown that these bound states are energetically favored even when they are localized, i.e., that the magnetization plateaus actually correspond to crystals of localized bound states rather than crystals of triplets^23^.

We model the Mg doping by introducing impurity sites where each impurity replaces one of the *S* = 1/2 spins on a dimer with a non-magnetic site (i.e., with no coupling to the neighboring sites), leaving a free *S* = 1/2 spin on the other site of the dimer. A single impurity in the lattice leads to a two-fold degenerate ground state, since it costs no energy to flip a single spin. Thus in the dilute limit of impurities we can expect that these free *S* = 1/2 spins immediately align with an external magnetic field. The question is now how the presence of these impurities with attached *S* = 1/2 spins affects the magnetization process, which we will investigate below using iPEPS simulations.

### iPEPS simulations results

To understand the impurity effects in the doped samples, the first key question is whether the bound states of triplets are effectively attracted or repelled by an impurity site and its neighboring *S* = 1/2 spin. To answer this question, we have performed simulations with a single impurity and one bound state in an 8 × 8 unit cell using a bond dimension *D* = 10, and found that the latter is clearly repelled by the impurity (see Supplementary Fig. 7). Thus, based on this result we can expect that in a large system containing many impurities, bound states are first created far away from neighboring impurities as magnetic field increases.

**Fig. 5:**
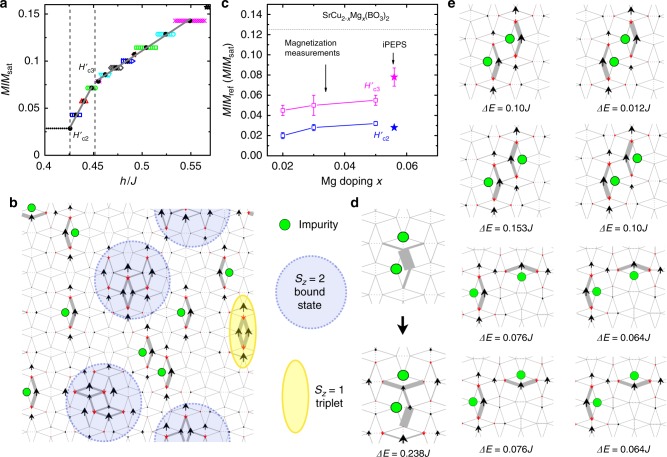
iPEPS simulation and magnetization measurements for the Mg doped SS model. **a** iPEPS magnetization curve obtained using a 12 × 12 unit cell with eight impurity sites. Black solid dots represent the centers of the plateaus. The full lines are linear fits to the black solid dots at high and low-field regions, respectively. Dashed lines indicate *H*’_C2_, and *H*’_C3_. A clear slope change is identified at *H*’_C3_. **b** Example spin configuration obtained in the total *S*_*z* _ = 11 sector. The size of the spins scale with the magnitude of the local magnetic moment, where black (red) arrows point along (opposite to) the external magnetic field. The thickness of the gray bonds scales with the local bond energy (the thicker the lower the energy). **c** Mg-doping dependence of the normalized magnetization, *M*/*M*_ref_, at *H*’_C2_ (magenta) and *H*’_C3_ (blue), obtained from experimental results on the *x* = 0.02, 0.03, and 0.05 samples, along with iPEPS simulation results for *M*/*M*_sat_ with an effective doping *x* = 0.056. Dashed line indicates the magnetization at the 1/8 plateau. The error bars of the magnetization measurements result from the uncertainty in determining magnetization values using the method shown in Supplementary Fig. 3. The iPEPS error bar takes into account the uncertainty of the location of *H*’_C3_ due to the finite unit cell size. **d** Special 2-impurity configuration in the *S*_*z*  _= 0 (top) and *S*_*z* _ = 1 (bottom) sectors, respectively, with an excitation energy ∆*E* = 0.238*J*. **e** Special 2-impurity configurations obtained with iPEPS which lead to additional excitation levels below *H*’_C1_ - at an onset field *H*’_C0_, as observed in the *x* = 0.05 sample

Figure 5a shows the iPEPS magnetization curve (*D* = 6) obtained using a 12 × 12 unit cell of dimers with a random configuration of eight impurity sites, corresponding to a doping *x* = 0.056. For this system, a localized bound state first occurs at *H*’_C2 _ = (0.428 ± 0.001)*J* (21.8 ± 0.1 T). In the infinite limit, since the system contains many locations in the lattice with similar energy costs to form a bound state, we may expect the non-smooth magnetization curve, exhibiting a jump at *H*’_C2_, compatible with the anomaly observed in experiments at *H*’_C2_ ~ 21.7 T (see Fig. 3). This value is essentially independent of the doping concentration, since a change of doping concentration only alters the density of such locations in the lattice, not the excitation energy. This is consistent with our experimental findings that the magnetization anomaly at *H*’_C2_ becomes stronger with increasing doping concentration, while the value of *H*’_C2_ is doping-independent. It is interesting to point out that *H*’_C2_ is close to the energy scale necessary to create a localized bound state in systems without impurities, 0.427*J*^23^. However, a localized bound state does not appear there, because its excitation energy (0.427*J*) is higher than that of a delocalized bound state, 0.41*J*^23^. Therefore, in undoped systems, bound states are delocalized in the dilute limit of excitations^42^, and a smooth increase of the magnetization, without a jump (anomaly) at *H*’_C2_, is expected.

Upon further increasing the magnetic field, the doped lattice gets occupied by more and more localized bound states. At a certain characteristic field *H*’_C3 _ = (0.452 ± 0.004)*J* (23.1 ± 0.2 T) we observe the appearance of additional triplet excitations in the lattice, accompanied by a rapid change of slope in the magnetization curve. This can be understood from the fact that a bound state occupies more space than a triplet excitation, so that at locations with several nearby impurity sites it can become energetically favorable to place a triplet excitation rather than a bound state. Thus, this suggests that the anomaly observed at *H*’_C3_ ~ 25 T in experiments is due to a rapid change of slope in the magnetization curve accompanied with the appearance of additional triplet excitations, rather than a magnetization plateau. Because the doping does not affect the lowest possible triplet excitation energy, *H*’_C3_ is also expected to be dependent only weakly on doping concentration. The probability to find such special locations in the lattice where it is preferable to put triplet excitations in the presence of impurities and bound states, however, increases with doping. Therefore, similar to our observation at *H*’_C2_, doping enhances the magnetization anomaly at *H*’_C3_ but does not change the value of *H*’_C3_. An example spin configuration at *H*’_C3_ is presented in Fig. 5b, containing three bound states and one triplet excitation. A good qualitative agreement between the simulation and experiments is demonstrated in Fig. 5c, which shows the doping dependence of the normalized magnetization *M*/*M*_ref_ at *H*’_C2_ and *H*’_C3_, extracted from the magnetization measurements (see Fig. 3) for the *x* = 0.02, 0.03, and 0.05 samples and the iPEPS simulations of *M*/*M*_sat_ for *x* = 0.056.

Finally, we address the additional features at *H*’_C1_ ~ 17.1 T and *H*’_C0_ ~ 9 T observed in experiments. As explained above, in the dilute limit of impurities we expect all the attached *S* = 1/2 moments to be aligned already at a small magnetic field. However, at larger doping there is an increasing probability of having two neighboring impurities, as shown in Fig. 5d. In this configuration, the *S* = 1/2 spins attached to the impurities can no longer be regarded as free, but they prefer to couple to a singlet. As a consequence, the two *S* = 1/2 spins do not immediately align with a small external field, but only do so beyond a certain critical field. From computing the excitation energy in a 8 × 8 cell we find a critical field *H*’_C1_ = 0.238*J* (~12.1 T), i.e. well below *H*’_C2_. This value corresponds to the excitation energy in the limit of an isolated pair of neighboring impurities. Here, the weak dependence of *H*’_C1_ on doping can be understood from the fact that changing the impurity concentration affects the probabilities of finding an (almost isolated) 2-impurity configuration, whereas its excitation energy remains unchanged—except at extremely high doping concentrations, which is beyond the scope of our current study. Therefore, the magnetization anomaly at *H*’_C1_ also becomes more prominent with increasing doping. In the presence of additional nearby impurities (e.g., a third impurity with an attached aligned spin in the vicinity of the impurity pair) the excitation energy will be higher, leading to a collaborative arrangement of impurities and additional energy excitation levels in between *H*’_C1_ and *H*’_C2_. As shown in Fig. 5e, there exist also other two-impurity configurations at lower excitation energies, which is consistent with the experimental observation of the broad maximum at an onset field *H*’_C0_ ~ 9 T. These further distant impurities have a smaller gap ∆*E*, with ∆*E* *→* 0 in the limit of large separations. The excitation energies are influenced also by additional impurities nearby. We note that the state at *H*’_C0_ is less prominent than the one at *H*’_C1_ = 0.238*J*, since the probability of these configurations to appear is smaller: e.g., the probability for the state with ∆*E* *=* 0.153*J* is only half of that at *H*’_C1_.

## Discussion

One of the most prominent properties of SrCu_2_(BO_3_)_2_ is the sequence of magnetization plateaus noticeably at 1/8, 1/4, and 1/3 of the saturation magnetization, which have been shown to correspond to various superstructures that break the translational symmetry of the lattice. It is remarkable that the high-field magnetization curve exhibits even more features in the presence of impurities. Impurities create local defects that are usually saturated by a small field. Our findings, however, portray a very different picture. Here, even a small concentration of impurity plays an important role in the magnetization process at very high magnetic fields. It highlights a nontrivial interplay between impurities, triplets, and bound states of triplets.

At high magnetic fields, the presence of impurities is found to disrupt the formation of superstructure of bound states of triplets and break it into small patches. In the *x* = 0.05 sample, these patches of superstructures account for ~85% of the total Cu^2+^ moments, as shown earlier. This significant softening of the superstructures is the reason for the smearing of the pseudo-1/*n* plateaus in the doped sample. It is expected that the pseudo-1/*n* plateaus would eventually be totally suppressed as the superstructures are further disrupted with increasing doping. Indeed, the fate of symmetry-breaking phases in the presence of impurities is itself of great current interest^52^. For the crystals of bound states formed at the magnetization plateaus of SrCu_2_(BO_3_)_2_^22,23^, the non-magnetic impurities generically break the symmetries of their order parameters and play the role of random field disorder^52^. Therefore, it is interesting to speculate if there exists impurity-induced Bragg glass phase analogous to that in a type II superconductor^53^. It is also intriguing for future studies to search for other novel phases such as Bose glass, which has been found near Bose-Einstein condensate phase in another doped quantum magnet^54^.

At fields lower than the pseudo-1/8 plateau, the emergence of the *H*’_C2_ and *H*’_C3_ anomalies upon doping is also a striking and unexpected result. In the undoped system, the energy cost for creating localized bound states is higher than that for the delocalized ones. Therefore, localized bound states are absent, and the bound states are delocalized in the dilute limit, leading to smoothly increasing magnetization curve (i.e., no anomaly at *H*’_C2_). In the doped system, however, the bound states cannot delocalize anymore due to the presence of the impurities. At *H*’_C2_ all the locations which are sufficiently far away from the impurities (and which have a similar energy cost to form a bound state) will be populated by a bound state, leading to a small jump in the magnetization (i.e., an anomaly) at *H*’_C2_. The *H*’_C3_ anomaly is also absent in the undoped case, where bound states form a superfluid in the dilute limit and regular crystals at higher density, i.e., localized triplets do not occur. Therefore, only in the presence of impurities, the *H*’_C2_ and *H*’_C3_ anomalies are realized. It is interesting to speculate how these states evolve, and if more exotic states with some special configurations of impurities and triplets would appear at higher doping.

The observation of the various 2-impurity configurations (impurity pairs) that survive up to *H*’_C0_ and *H*’_C1_ is another significant result. It is found that the excitation energies for these impurity pairs are doping independent, at least in the doping range of our study, but the probability of finding them increases with doping. Our results demonstrate a rich and interesting geometrical arrangement of impurities in the doped SrCu_2_(BO_3_)_2_. Moreover, these spin-singlet impurity pairs are fairly stable (binding energy ≳ *gµ*_B_*H*’_C0_ ~ 1 meV), and it is interesting to speculate the formation of Cooper pairs if holes (or electrons) can be associated with the impurities without too much energy cost. Note that the impurity pairs that we observe are strongly localized, suggesting that Cooper pairs, if present, might also be strongly localized. It is interesting to note that in cuprates as well as the doped SrCu_2_(BO_3_)_2_ studied in ref. ^11^, the impurities reside in between the copper-oxide planes, while in SrCu_2−*x*_Mg_*x*_(BO_3_)_2_, Mg^2+^ replaces Cu^2+^ in the CuO_4_ plane. Further studies are required to explore the difference between the impurity configurations in the two cases.

In summary, our results present the Mg-doped SrCu_2_(BO_3_)_2_ as a model system for studying the effects of non-magnetic impurities in a frustrated quantum magnet, where results from theoretical simulation and multiple experimental methods can be directly compared. We have provided a clear description of the magnetization process for a Shastry-Sutherland system in the presence of impurities, which has a profound effect on the formation of the crystals of bound states of triplets. The results also reveal a rich impurity-induced phenomenology at fields below the magnetization plateaus, suggesting that even for samples with a Mg-doping as low as 1% ∼ 2.5%, a single-impurity description such as that discussed in refs. ^15,16^ is not enough to capture the essential physics, and interactions between the impurities and triplets must be considered. Further studies are desired to better understand the impurity-induced emergent states, to search for other possible novel phases (Bragg glass, Bose glass) at high fields, and to pursue the grand prize of RVB superconductivity.

## Methods

### Sample synthesis and characterization

High quality single crystal samples of both SrCu_2_(BO_3_)_2_ and SrCu_2−*x*_Mg_*x*_(BO_3_)_2_ (*x* = 0.02, 0.03, and 0.05) were grown by the optical floating zone technique using self-flux, at a growth rate of 0.2 mm h^−1^ in an O_2_ atmosphere^55^. The *x* = 0.02 and 0.03 samples were successfully grown and were characterized by X-ray powder diffraction. The free *S* = 1/2 impurities, i.e., the Mg-doping concentrations, for the *x* = 0.02 and 0.03 samples were also characterized with measurements of the dc susceptibility as a function of temperature, using a commercial Quantum Design MPMS (see Supplementary Fig. 1).

### Magnetization measurements

Magnetization measurements were conducted on samples with approximate dimensions of ∼3.0 × 0.5 × 0.5 mm^3^ (*a* *×* *b* *×* *c*) using a sample-extraction magnetometer in a 25 ms, 65 T pulsed magnet at the pulsed field facility of the National High Magnetic Field Laboratory in Los Alamos, NM^24^. The sample was placed inside a plastic capsule, which is inserted into or extracted from a pair of coaxial counterwound coils. The background signal was also determined for each temperature and subtracted from the data. Data was obtained for **H** ∥ *a*-axis down to 0.4 K, and calibrated with absolute values measured in a SQUID magnetometer from Quantum Design.

### Tunnel diode oscillator

The TDO measurements^56^ were carried out on cylinder-shaped crystals with approximate dimensions of ~2 mm in length and ~1 mm in diameter, at the dc field facility of the National High Magnetic Field Laboratory in Tallahassee, FL. A tunnel diode, operating in its negative resistance region, was used to provide power that maintains the resonance of a LC-circuit, at a frequency range between 10 and 50 MHz. The sample was placed inside a detection coil, with the *a*-axis of the sample aligned with the coil axis, forming the inductive component of the LC circuit. Changes in sample magnetization induce a change in the inductance, which is detected as a shift in the resonance frequency. The ability to measure the resonance frequency to a very high precision ensures the highly sensitive measurements in changes of magnetic moments ~ 10^−12^ e.m.u.^57^.

### Torque magnetometry

Torque magnetometry measurements were conducted to probe the susceptibility anisotropy of samples with approximate dimensions of ~0.2 × 0.2 × 0.2 mm^3^ (*a* *×* *b* *×* *c*) in static magnetic field^22^. Samples were attached with silicone grease to the commercial piezoresistive atomic force microscopy (AFM) cantilevers (Seiko PRC400)^58^, which form a Wheatstone bridge configuration with two additional adjustable resistors. Changes of sample magnetization with field induced torque on the cantilever, and are detected as a voltage across the bridge.

### Magnetostriction measurements

An optical fiber, equipped with a 1-mm-long fiber Bragg grating (FBG), was attached to single crystal samples with approximate dimensions of ~ 3.0 × 0.5 × 0.5 mm^3^ (*a* *×* *b* *×* *c*) along their *a*-axes, using cyanoacrylate. The samples were held in place solely by the fiber, and were orientated such that the applied field is parallel with the *a*-axes of the samples. The FBG is illuminated by a broadband light (1525–1565 nm) source, and reflects a narrow band of light (≈1550 nm)^59^. The length variation ∆*L*/*L* along *a*-axis axial configuration is detected by monitoring the shift of the reflected light by the FBG^20^.

## Supplementary information

Supplementary Information

Peer Review

## Data Availability

The data that support the findings of this study are available from the corresponding author upon reasonable request.
