# Supplementary material for: Emergent bound states and impurity pairs in chemically doped Shastry-Sutherland system
Source: Nat Commun. 2019 Jun 4;10:2439. doi: 10.1038/s41467-019-10410-x (PMC6547672; doi:10.1038/s41467-019-10410-x)
Supplement: Supplementary file 1 — Supplementary Information [file 41467_2019_10410_MOESM1_ESM.pdf]

Supplementary Material for: Emergent Bound States and Impurity Pairs in Chemically Doped Shastry-Sutherland System

Shi et al.

Supplementary Note 1: Data reproduced from previous magnetization measurements on the undoped sample

The magnetization measurements in Ref. 1 (adapted with permission, copyright American Physical Society 2005) were performed on the undoped (x = 0) sample with H || c, and a background 0.14×10^3 emu per Cu mol, which was attributed to crystalline defects, was subtracted. In our Mg-doped samples, however, impurities due to crystalline defects could not be differentiated from the Mg-induced spin impurities. Therefore, the background was added back to the reproduced x = 0 data from Ref. 1, to allow a fair comparison. Moreover, in the reproduced x = 0 data, $\mu_0 H$ is multiplied by the g-factor ratio $g_{\parallel c}/g_{\parallel a}$ = 2.28/2.04 = 1.12 (Ref. 2), and M is divided by the same g-factor ratio, to account for the different field orientations.

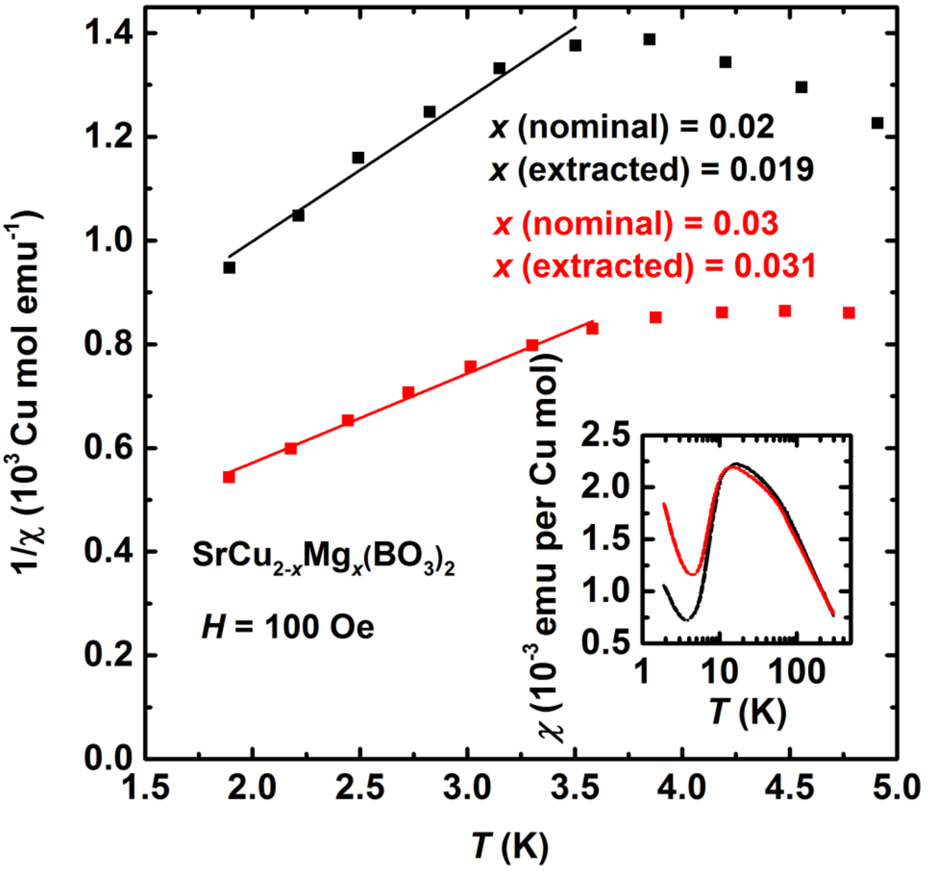

Supplementary Figure 1: **Temperature dependence of the inverse magnetic susceptibility.** Data are shown for the x = 0.02,0.03 samples in an applied field of 100 Oe, parallel to the ab plane**.** The fits to the Curie-Weiss law $C'/(T-\theta')$ in the 1.8 K - 3.5 K regime, as shown by the solid lines, give C'(low-T) = 3.6 × 10^{-3} emu K per Cu mol and 5.8 × 10^{-3} emu K per Cu mol for the x = 0.02 (black) and x = 0.03 (red) samples respectively, which correspond to x = 0.019 and x = 0.031, assuming free S = 1/2 impurity spins. (Inset) $\chi(T)$ for the entire temperature range (1.8 K ≤ T ≤ 300 K).

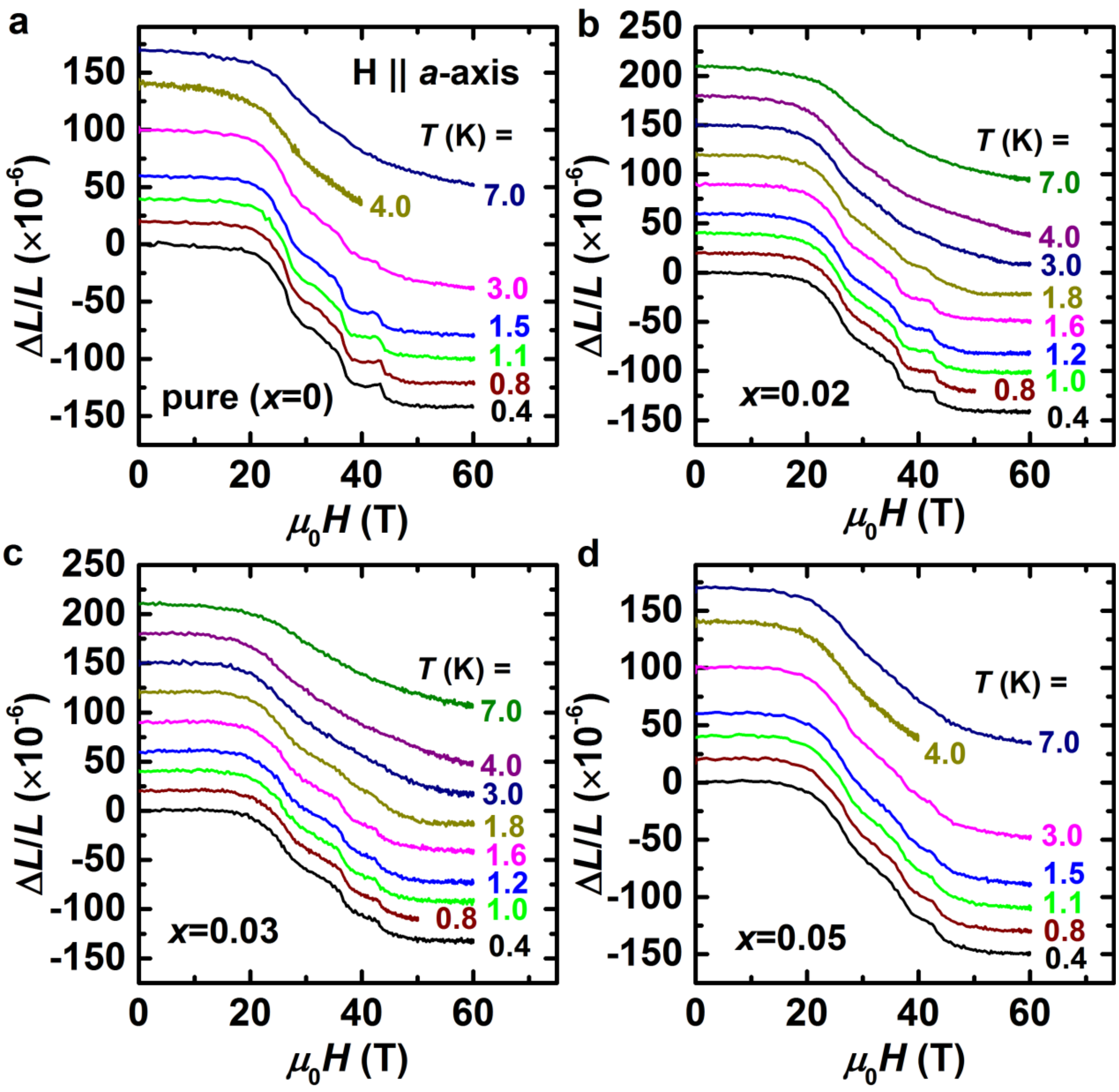

Supplementary Figure 2: **Temperature evolution of the plateaus in magnetostriction measurements.** Data are shown for (a) the undoped (x = 0) and (b − d) the Mg-doped (x = 0.02,0.03,0.05) samples. Field (**H** ∥ a-axis) dependence of $\Delta L/L$ in pulsed fields up to 60 T. Data presented was taken during field upsweep. Traces are shifted for clarity.

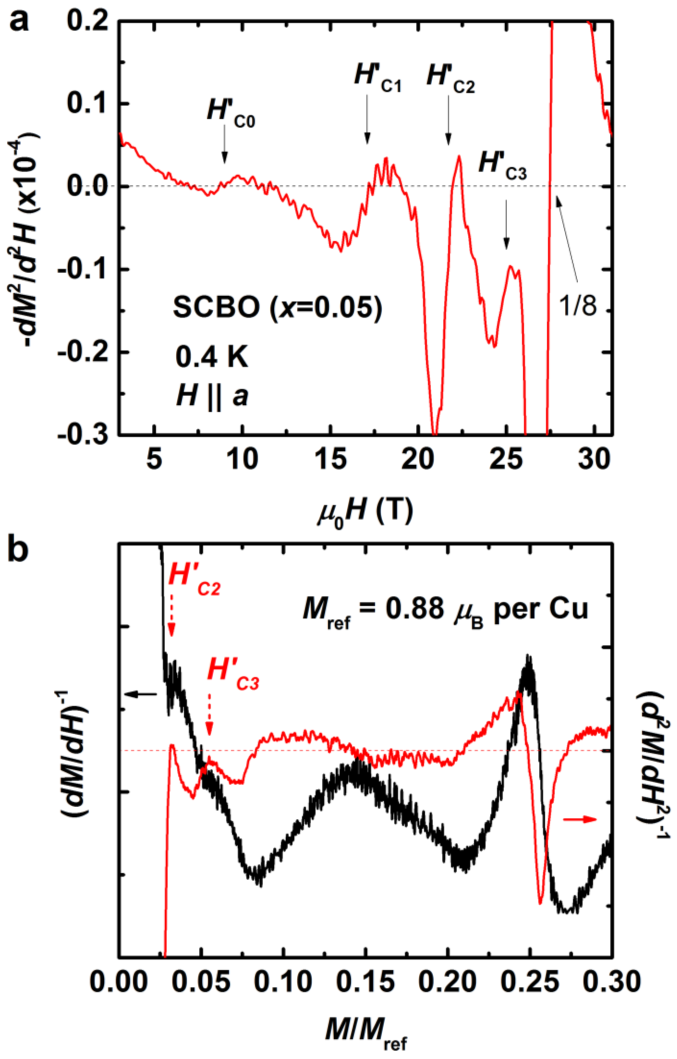

Supplementary Figure 3: **Method to determine the onset magnetic fields and magnetization values of the anomalies.** Data are shown for the x = 0.05 sample at T = 0.4 K. **a**, H'_{C0}, H'_{C1}, and H'_{C2} are defined as the fields where $-d^2M/dH^2$ crosses zero from below, i.e., peaks in dM/dH vs. H. H'_{C3} is defined as the peak in $-d^2M/dH^2$ vs. H. **b**, The inverse susceptibility $(dM/dH)^{-1}$ (black curve, left axis) and the inverse second derivative $(d^2M/dH^2)^{-1}$ (red curve, right axis) vs. M/M_{ref}. The magnetization values M/M_{ref} at H'_{C2} and H'_{C3} are determined as the maxima in the inverse second derivative, as shown with the red dashed arrows. The analyses were repeated for the x = 0.02 and 0.03 samples.

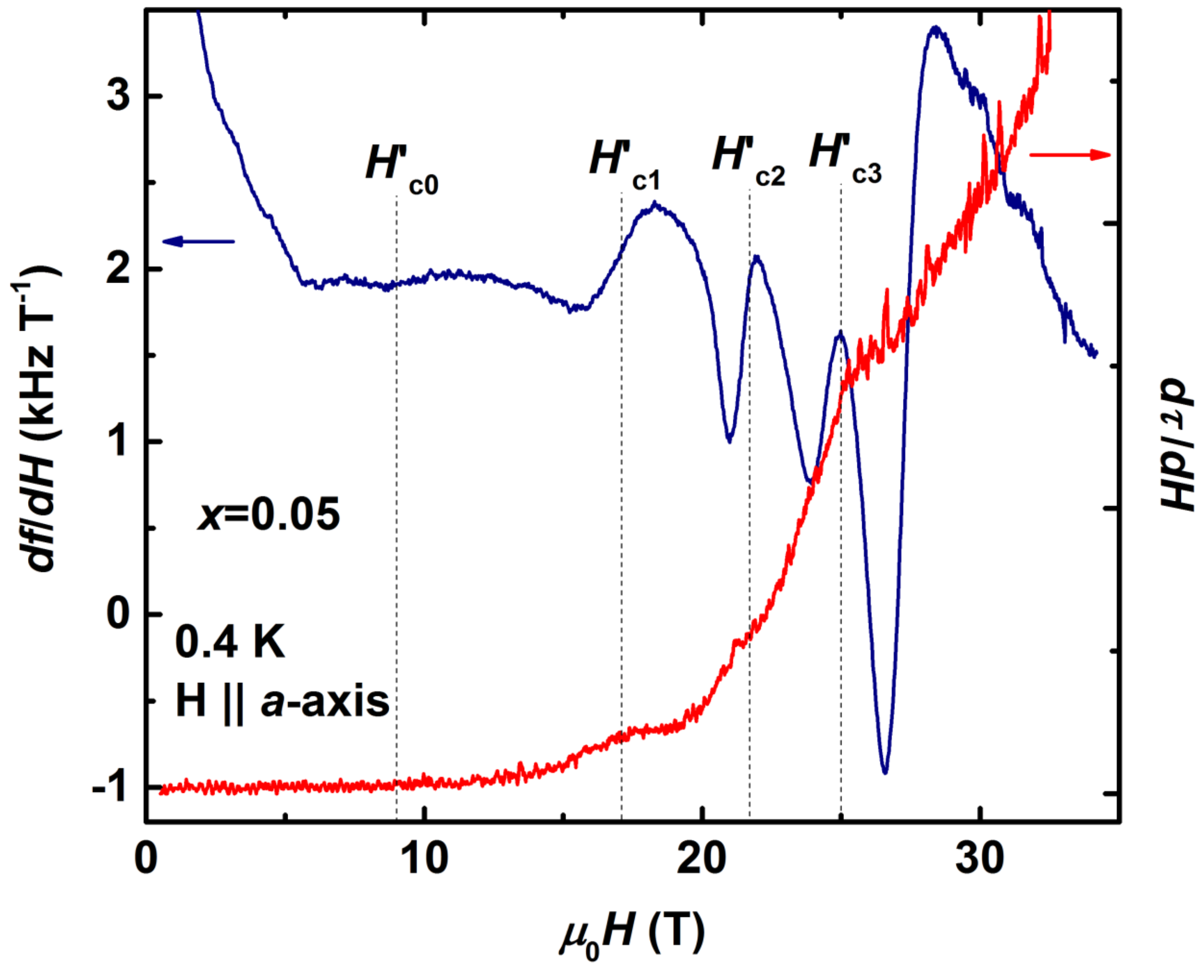

Supplementary Figure 4: **The TDO and torque magnetometry measurements for the *x* = 0.05 sample.** Field (**H** ∥ *a*-axis) dependence of df/dH ($\propto dM^2/d^2H$) from the TDO measurements (blue, left axis), and $d\tau/dH$ from the torque magnetometry measurements (red, right axis) at 0.4 K. The H'_{C0}, H'_{C1}, H'_{C2}, and H'_{C3} anomalies, identified from the TDO and magnetization measurements are indicated by the dashed lines. The latter three anomalies also appear in the torque magnetometry measurements. Measurements were repeated for the *x* = 0.02 and *x* = 0.03 samples, and similar results were obtained.

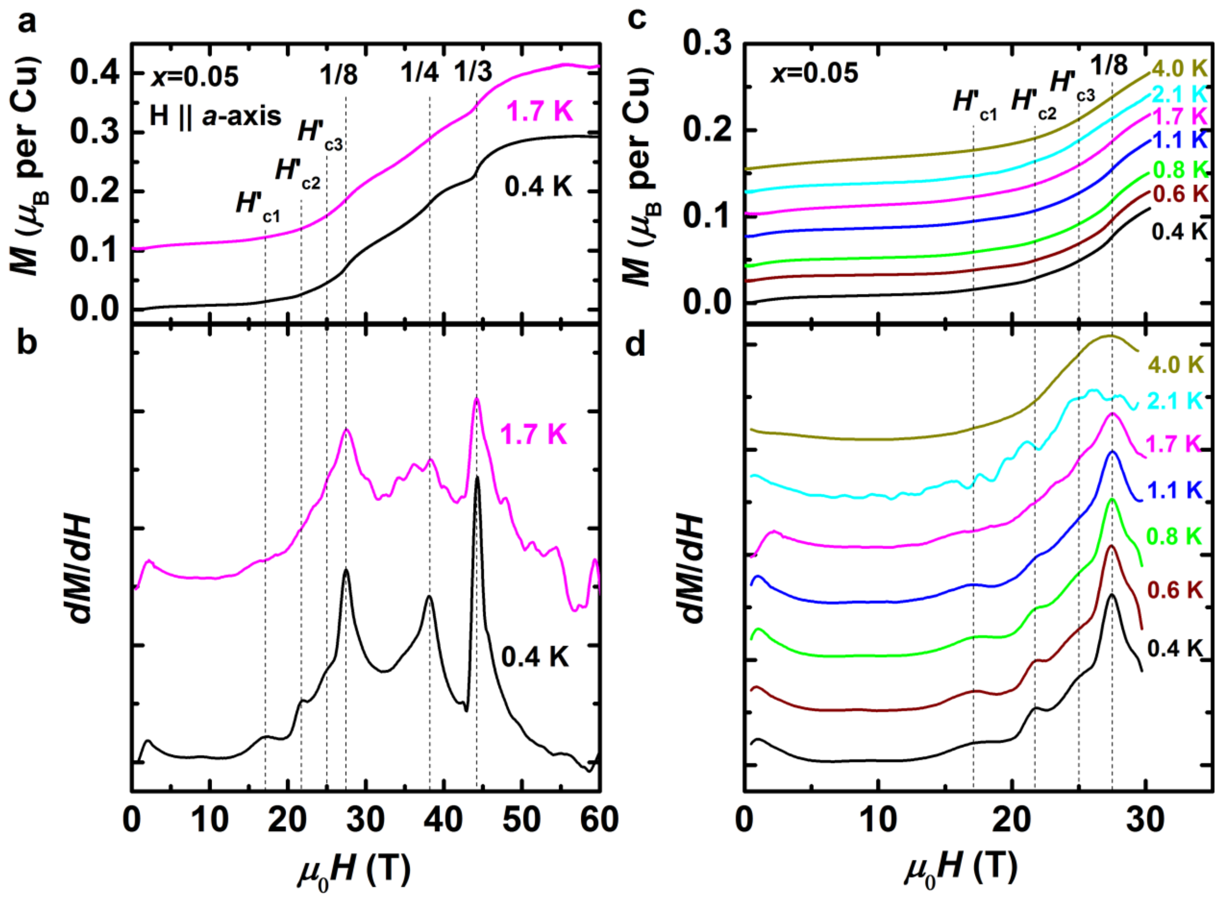

Supplementary Figure 5: **Evolution of the H'_{C1}, H'_{C2} and H'_{C3} anomalies and the pseudo plateaus with temperature for the x = 0.05 sample.** Field (**H** ∥ a-axis) dependence of (**a** and **c**) M and (**b** and **d**) dM/dH in pulsed fields up to 60 T and 30 T, for temperatures from 0.4 K (black), to 1.7 K (magenta), to 4.0 K (dark yellow). The H'_{C1}, H'_{C2} and H'_{C3} anomalies and the plateaus are indicated by the dashed lines. Data presented was taken during field upsweep. Traces are shifted for clarity.

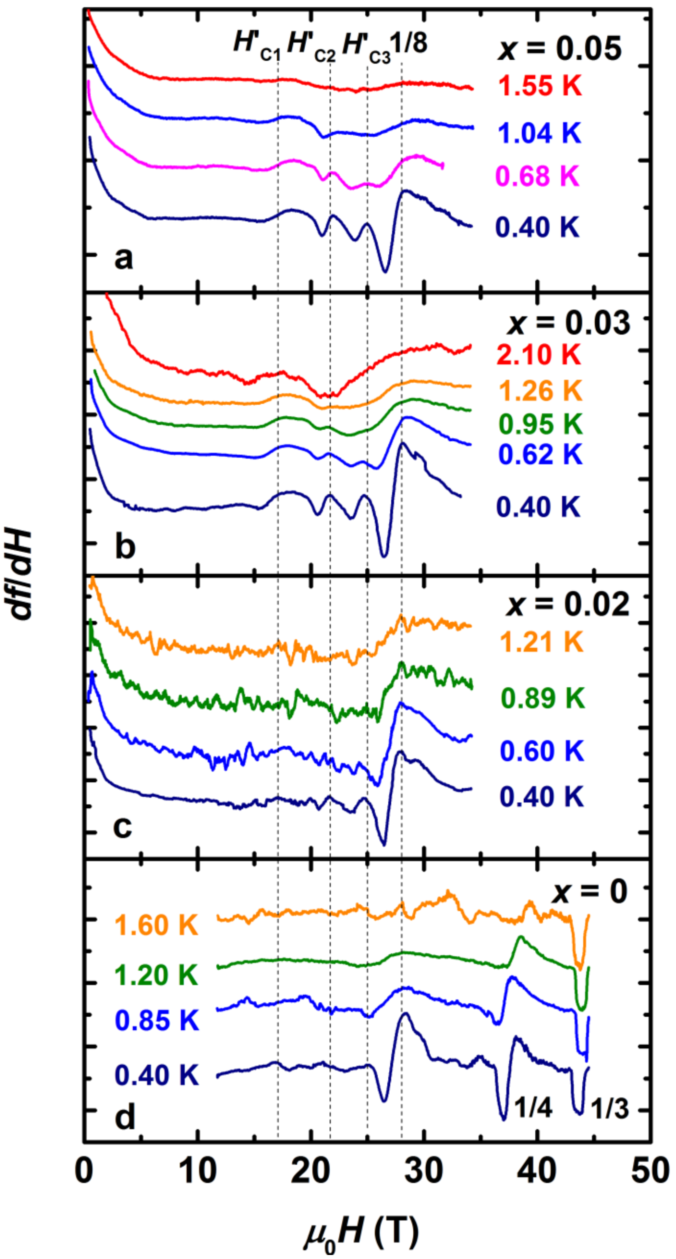

Supplementary Figure 6: **Evolution of the H'_{C1}, H'_{C2} and H'_{C3} anomalies and the (pseudo-) 1/8 plateau with temperature in the TDO measurements.** Data are shown for (a – c) the Mg-doped (x = 0.05, 0.03, 0.02) and (d) the undoped (x = 0) samples. Field (**H** ∥ a-axis) dependence of df/dH ($\propto dM^2/d^2H$) in static fields. Traces are shifted for clarity. Dashed lines guide the eye.

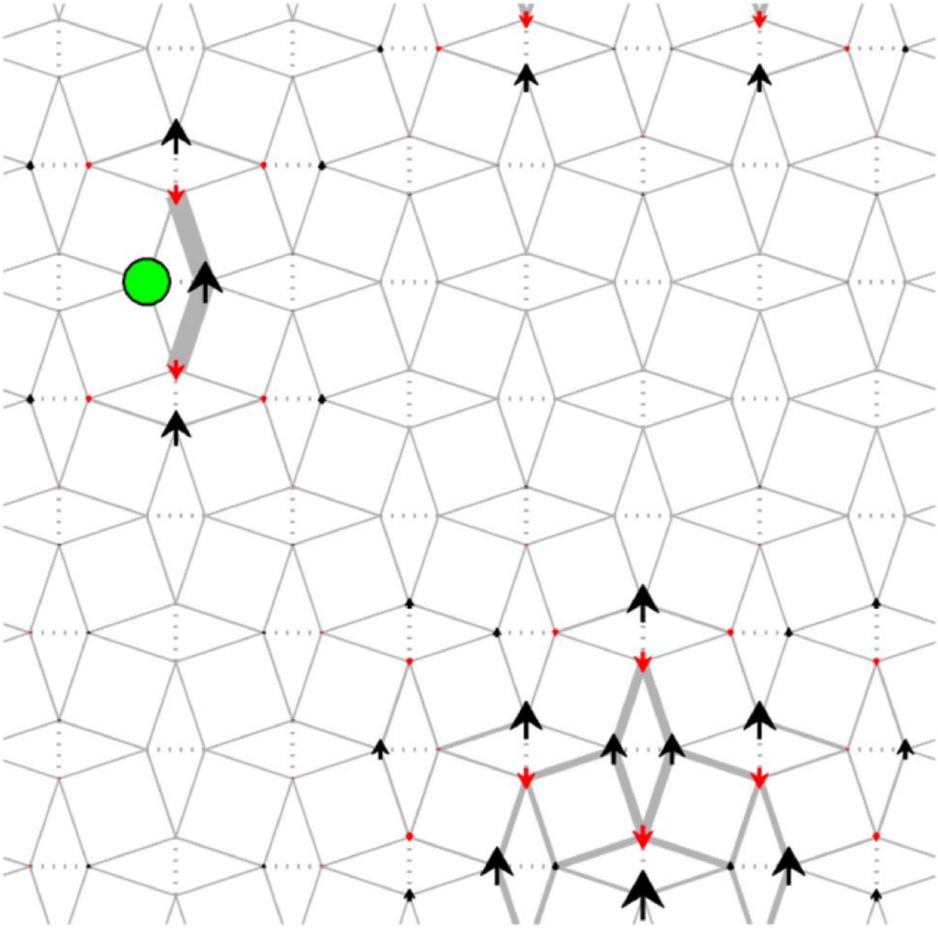

Supplementary Figure 7: **iPEPS results for lowest energy state in an 8×8 unit cell.** Simulations periodically repeated in the infinite lattice; including one impurity (green disc) with a neighboring S=1/2 site and one S_z = 2 bound state (partially delocalized) away from the impurity, showing that a bound state is not attracted but repelled by an impurity site. The size of the spins scale with the magnitude of the local magnetic moment, where black (red) arrows point along (opposite to) the external magnetic field. The thickness of the grey bonds scales with the local bond energy (the thicker the lower the energy).

Supplementary References:

1. Jorge, G. A., *et al.* Crystal symmetry and high-magnetic-field specific heat of $SrCu_2(BO_3)_2$. *Phys. Rev. B* **71**, 092403 (2005).
2. Jaime, M., *et al.* Magnetostriction and magnetic texture to 100.75 Tesla in frustrated $SrCu_2(BO_3)_2$. *Proc. Natl. Acad. Sci. USA* **109**, 12404–12407 (2012).
