# Supplementary material for: Emergent bound states and impurity pairs in chemically doped Shastry-Sutherland system
Source: Nat Commun. 2019 Jun 4;10:2439. doi: 10.1038/s41467-019-10410-x (PMC6547672; doi:10.1038/s41467-019-10410-x)
Supplement: Supplementary file 2 — Peer Review [file 41467_2019_10410_MOESM2_ESM.pdf]

Reviewers' comments:

Reviewer #1 (Remarks to the Author):

The manuscript by Shi and coauthors present magnetic properties measurements in low temperature and high field, as well as theoretical simulations on Mg doped Shastry-Sutherland compounds SrCu2(BO3)2. The main observation is that below the field for 1/8 magnetization plateau, they found 4 magnetization anomalies at Hc0, Hc1, Hc2, and Hc3, respectively. Combining with the iPEPS simulations, the authors interpret Hc0 and Hc1 corresponding to the fields to align the s=1/2 spins at some two-impurity configurations, Hc2 to the critical field to create a localized bound states of triplets, and Hc3 to the change slope in the magnetization when the lattice occupied by additional triplets excitations. These results indicate that the impurities induced by Mg doping interacts strongly with both triplets and bound states of triplets.

The experimental finding of 4 magnetization anomalies is novel and interesting. The interpretation of the origins of anomalies is reasonable. The manuscript would be of interest to those working in the field. I believe this paper is suitable for publication in Nature Communications after the authors consider my following concern.

The experimental results in fig. 5c show that Hc1, Hc2, and Hc3 are weak dependent of doping concentration x. In the manuscript, the authors carried out simulations only with an effective doping x=0.056. I suggest the authors to do simulations with low doping level to verify these fields are weak dependent of x.

Reviewer #2 (Remarks to the Author):

Please download the attached file.

Reviewer #3 (Remarks to the Author):

In this paper, the authors report unexpected anomalies in the magnetization curve of doped SrCu2(BO3)2, also providing numerical calculations based upon the Heisenberg model on the Shastry-Sutherland lattice.

The paper is interesting but I have a few questions:

1) First of all, I do not understand the definition of M_{ref}=3*M_{1/3}. The authors say that "M_{1/3} refers to the magnetization at the 1/3 plateau", which should be 1/3 by definition, for all dopings. So M_{ref}=1 always.

2) In the inset of Fig.1a, the curve for x=0.02 is above the one for x=0.03 for H<2. Is it clear the reason for this behavior?

3) Similarly, I am a bit surprised about the non-monotonic behavir of the magnetization curve of Fig.2, especially when considering the undoped case with x=0.

4) In Fig.3a, for H<25, the curves for x=0.03 and 0.05 almost coincide, while the one for x=0.02 is much below, suggesting a saturation when x>0.03. Do the authors have an explanation for that? It would be also nice to investigate this issue by using iPEPS.

5) The setup of iPEPS is such that the disorder is not a real disorder (the 12x12 unit cell is periodically

repeated), I am wondering how much the estimations of H_{c2} and H_{c3} depend on the disorder configuration that has been chosen.
I would be happy to have an errorbar on the determination of these critical fields: for example the magnetization curve obtained within iPEPS is not smooth (Fig.5a), and determining the change of slope can be affected by the fitting procedure: to be honest, I can even trace a single line (i.e., without visible anomaly) fitting the iPEPS data.

Moreover, it is written that "this suggests that the anomaly observed at $H_{c3} \sim 25T$ in experiments is due to a change of slope in the magnetization curve". I find this claim quite strange, since, by looking at the experimental data, it should be easy to see if there is a change in the slope or not. May the authors be more precise on that?

6) In addition, it is claimed that the value of H_{c2} obtained by iPEPS with disorder is not very different from the one without disorder. However, for $x=0$, the experimental data do not show a significant singularity at H_{c2}. Therefore, I am not sure if the way to determine H_{c2} within iPEPS is correct or not.

From iPEPS, H_{c2} is associated to the existence of a localized bound state: I am wondering if this statement can be confirmed within the experimental setup.

Minor points:

1) Why is the acronym of the Shastry-Sutherland model SSL and not just SS?

2) I think that in the introductory part too much emphasis is put on the possibility to have superconductivity. This aspect is instead not addressed in the paper. I suggest to smooth these statements.

In summary, I think that the paper can be published, but only after the authors will address the points raised above.

Reply to the Reviewers

We are grateful to the Reviewers for their constructive suggestions on content and clarity. To address the Reviewers' comments, we have performed substantial modifications and revised the manuscript accordingly. We have attempted to address all the concerns raised by making the following changes (comments in blue, replies to comments in black):

Reviewer #1:

The manuscript by Shi and coauthors present magnetic properties measurements in low temperature and high field, as well as theoretical simulations on Mg doped Shastry-Sutherland compounds SrCu2(BO3)2. The main observation is that below the field for 1/8 magnetization plateau, they found 4 magnetization anomalies at Hc0, Hc1, Hc2, and Hc3, respectively. Combining with the iPEPS simulations, the authors interpret Hc0 and Hc1 corresponding to the fields to align the s=1/2 spins at some two-impurity configurations, Hc2 to the critical field to create a localized bound states of triplets, and Hc3 to the change slope in the magnetization when the lattice occupied by additional triplets excitations. These results indicate that the impurities induced by Mg doping interacts strongly with both triplets and bound states of triplets.

The experimental finding of 4 magnetization anomalies is novel and interesting. The interpretation of the origins of anomalies is reasonable. The manuscript would be of interest to those working in the field. I believe this paper is suitable for publication in Nature Communications after the authors consider my following concern.

The experimental results in fig. 5c show that Hc1, Hc2, and Hc3 are weak dependent of doping concentration x. In the manuscript, the authors carried out simulations only with an effective doping x=0.056. I suggest the authors to do simulations with low doping level to verify these fields are weak dependent of x.

We thank the Reviewer for the the positive assessment and constructive suggestion. We agree with the Reviewer that it would be useful to discuss why the critical fields depend only weakly on the doping concentration *x* based on the iPEPS simulations. Therefore, performing additional simulations as function of chemical doping would be valuable follow-up endeavors, however, adding such results to the current manuscript would be a substantial undertaking, computationally demanding and time consuming. To that end, we have added the necessary discussion to the manuscript to clarify this point and address the Reviewer's concern based on our present simulation results. Below please see the detailed corresponding descriptions:

Considering that H_{c1} is related to a special 2-impurity configuration, in an infinite system this configuration, along with its associated excitation energy, will always appear with a finite probability. This probability depends on the doping concentration *x* - the larger the *x* value, the larger the probability to have two nearby impurities. However, the associated excitation energy (i.e. the value of H_{c1}) is not affected by the change in the *x* value – that is except in case of extremely high doping concentrations, which are not subject of this study and are not considered here. The reason is that in case of low doping concentrations, the special 2-impurity configurations

are statistically located far away from other impurities, and therefore their excitation energy is insensitive to small changes in doping.

A similar argument can also be made for H_{c2}: for any doping concentration *x,* there is a certain probability to find locations in the lattice, which are sufficiently far away from the nearest impurities, such that the excitation energy to form a bound state at these locations is comparable to the one of a localized bound state without impurities. Therefore, changing the doping concentration only affects the number of such locations in the lattice, but the value of the excitation energy (i.e. the value of Hc_2) is not affected.

For similar reasons, we do not expect a strong dependence of H_{c3} on x, either. At H_{c3}, there exists special locations in the lattice, where it is preferable to put triplet excitations in the presence of impurities and bound states. Such special locations can be found for any x, and the value of x will affect this probability, but not the excitation energy itself.

In order to clarify this issue, we have expanded the discussion in the sections of "iPEPS simulation results" and "Discussion".

a) On page 12, the following discussion regarding H'_{C2} is added:

"For this system, a localized bound state first occurs at H'_{C2} = (0.428 ± 0.001)J (~ 21.8 ± 0.1 T). In the infinite limit, since the system will contain many locations in the lattice with similar energy costs to form a bound state, we may expect that the magnetization curve is not smooth but that it exhibits a jump at H'_{C2}, compatible with the anomaly observed in experiments at H'_{C2} ~ 21.7 T (see Fig. 3). This value is essentially independent of the doping since a change of doping only alters the density of such locations in the lattice, but not the excitation energy. This is consistent with our experimental findings that the magnetization anomaly at H'_{C2} becomes stronger with increasing doping while the value of H'_{C2} is doping-independent."

b) On page 13, the following discussion regarding H'_{C3} is added:

"Because the doping does not affect the lowest possible triplet excitation energy, H'_{C3} is also expected to be dependent only weakly on doping. The probability to find such special locations in the lattice where it is preferable to put triplet excitations in the presence of impurities and bound states, however, increases with doping. Therefore, similar to our observation at H'_{C2}, doping enhances the magnetization anomaly at H'_{C3} but does not change the value of H'_{C3}."

c) On page 14, the following discussion regarding H'_{C1} is added:

"Here, the weak dependence of H'_{C1} on doping can be understood from the fact that changing the impurity concentration affects the probabilities of finding an (almost isolated) 2-impurity configuration but not its excitation energy, except at extremely high doping concentration which is beyond the scope of our current study. Therefore, the magnetization anomaly at H'_{C1} also becomes more prominent with increasing doping."

d) On page 16, in the "Discussion" section, the following sentences are added:

"It is found that the excitation energies for these impurity pairs are doping independent, at least in the doping range of our study, but the probability for finding them increases with doping."

In summary, we have made extensive changes in the revised paper to clarify the reasons for the weak doping dependence of H'_{C1}, H'_{C2}, and H'_{C3}, which is the Reviewer's main concern. We believe that these modifications should clarify this important point, and thank the Reviewer for his/her helpful comments.

Reviewer #2

In this manuscript authors studied the magnetic response of Mg-doped SrCu2(BO2)3 single crystals under high magnetic field using different magnetometry techniques. Expectedly, authors observed magnetization plateaus at 1/3, 1/4 and 1/8 of the saturation magnetization for all undoped and doped samples, which shows that doping is not affecting much the spin state of the system even at higher magnetic fields. The most interesting part of this manuscript is the observation of three anomalous magnetization transitions (states) at $H'c1$, $H'c2$, and $H'c3$ at lower fields, below 1/8 plateau. However, the origin of these transition is not completely clear.

We thank the Reviewer for her/his time reviewing our manuscript, and for welcoming the results we have reported in our paper. We agree with the Reviewer that an improved presentation of our results is needed to clarify the origin of the H'_{C1}, H'_{C2}, and H'_{C3} anomalies. We have thus made extensive changes to the manuscript to improve our presentation, as discussed below.

$H'c2$ is related with localized bound states, which is also close to the field at which bound state created without impurities. Does it mean impurities just enhancing the magnetization jump at $H'c2$? $H'c3$ where triplet population enhanced, which is not expected for an undoped sample?

We are grateful to the Reviewer for pointing out the need to clarify these questions and to address the confusion regarding the comparisons between the doped and undoped cases. Therefore, in the revised manuscript, we have carefully modified the corresponding discussion, and explicitly explained in detail the effects of impurities on H'_{C2}, and H'_{C3}. Here, we will first clarify this issue, and then summarize the corresponding changes we have made in the revised manuscript.

In absence of impurities, the bound states are actually delocalized in the dilute limit of excitations, and a smooth increase of the magnetization (i.e. No jump/anomaly) is expected at H'_{C2}. The statement we made in the paper is that H'_{C2} is close to the field which is necessary to create a *localized* bound state with no impurities - as computed in Ref. 23. However, as reported in Ref. 23, in absence of impurities, the excitation energy of the *localized* bound state is higher than the that of a *delocalized* bound state, being the ground state in the dilute limit of excitations. In the doped samples, however, the *localized* bound states are stabilized, and probability of finding them increases with doping concentration x - though their excitation energy is only weakly dependent on doping, as discussed in details in our response to Reviewer 1's comment. Therefore, the H'_{C2} anomaly associated with the localized bound states is only expected in the doped samples, and not in the undoped sample.

Regarding H'_{C3}, the Reviewer is correct that it is not expected for an undoped sample. This is because in the undoped case the bound states form a superfluid in the dilute limit and regular crystals at higher density, i.e. *localized* triplets do not occur in the undoped sample. Therefore, both H'_{C2} and H'_{C3} anomalies are emergent states that only appear in the doped samples.

In response to the Reviewer's comment, we have made the following changes:

a) We have added a sentence on page 9 of the revised manuscript, and it reads:

"This is consistent with the fact that these anomalies were never observed in the undoped system[19,21,24]."

b) At the bottom of page 12, we have added the following sentences, also in response to a relevant comment by Reviewer 3:

"It is interesting to point out that H'_{C2} is close to the energy scale necessary to create a localized bound state in systems without impurities, 0.427J (Ref. 23). However, a localized bound state does not appear there because its excitation energy (0.427J) is higher than that of a delocalized bound state, 0.41J (Ref. 23). Therefore, in undoped systems, bound states are delocalized in the dilute limit of excitations[42], and a smooth increase of the magnetization, without a jump (anomaly) at H'_{C2}, is expected."

c) On page 13, the beginning of the second paragraph is rewritten as following:

"Upon further increasing the magnetic field, the doped lattice gets occupied by more and more localized bound states. At a certain characteristic field $H'_{C3} = (0.452 \pm 0.004)J$ (~ 23.1 ± 0.2 T) we observe the appearance of additional triplet excitations in the lattice, accompanied by a rapid change of slope in the magnetization curve."

d) In response also to a relevant comment by Reviewer 3, and to avoid confusions we have clarified the comparison between the doped and undoped cases in the "Discussion" section by replacing the previous text with the following sentences on page 16:

"In the undoped system, the energy cost for creating localized bound states is higher than that for the delocalized ones. Therefore, localized bound states are absent, and the bound states are delocalized in the dilute limit, leading to smoothly increasing magnetization curve (i.e. no anomaly at H'_{C2}). In the doped system, however, the bound states cannot delocalize anymore due to the presence of the impurities. At H'_{C2} all the locations which are sufficiently far away from the impurities (and which have a similar energy cost to form a bound state) will be populated by a bound state, leading to a small jump in the magnetization (i.e. an anomaly) at H'_{C2}. The H'_{C3} anomaly is also absent in the undoped case, where bound states form a superfluid in the dilute limit and regular crystals at higher density, i.e. localized triplets do not occur. Therefore, only in the presence of impurities, the H'_{C2} and H'_{C3} anomalies are realized."

It will be good to show the derivative of magnetization (dM/dH) for $x = 0$ as well.

We thank the Reviewer for the constructive advice, and we agree that it is indeed informative to carefully examine the existence of such anomalies (H'_{C0}, H'_{C1}, H'_{C2} and H'_{C3}) also in the $x = 0$ sample. We note that in this study we only measured magnetization for *doped* samples and the $x = 0$ magnetization data shown in Fig. 2 has been previously reported in Ref. 24, however, this information can also be extracted from our TDO data shown in Fig. 3c. We show in Fig. 3d that the df/dH obtained from the TDO measurements is proportional to the second derivative of magnetization dM^2/d^2H. Therefore, since our TDO data collected on the doped samples represents higher order derivative of the magnetization with respect to field, it shows the same anomalies as those observed in dM/dH with even higher resolution. Also in case of the undoped sample ($x = 0$), it is clearly seen that no anomaly is observed at H'_{C0}, H'_{C1}, H'_{C2} and H'_{C3}, as expected – please see the discussions above regarding H'_{C2} and H'_{C3}, and notes below regarding H'_{C0} and H'_{C1}. We note that our TDO data was obtained in a steady magnetic field and our magnetization data was acquired using pulsed field, and considering the difference in experimental setup we find the agreement between these two data sets (Fig. 3d) remarkable.

With increasing doping ½ spin is not anymore free because of neighbouring impurities. Thus, they do not align at lower field but at higher critical field $H'c1$. It means $H'c1$should strongly dependent on the doping concentration? However, in the present experiments it does not happen. How authors discuss the insensitivity of anomalous magnetization jumps ($H'c1$, $H'c2$, and $H'c3$) again the doping concentration.

We appreciate the Reviewer's interesting observation, and we agree that this point needs to be discussed further in the paper. In fact, Reviewer 1 has raised the very same point, therefore, please see our detailed reply to these questions, as well as the changes that we have made in the manuscript to address them on page 1-3 of this reply.

Theoretically, it has been predicted that s-wave and/or d-wave superconductivity can be emerged in doped SrCu2(BO2)3. It means it is desirable to study the effects of the doping and emergence of the superconductivity. However, this work does not show any evidence of induced superconductivity and origin of anomalous magnetization transitions is also not clear.

We are thankful to the Reviewer for appreciating the relevance of our study to the broader efforts in the community to search for superconductivity in this material. As the Reviewer pointed out, our work actually focuses on the effects of doped impurities on the magnetization process in this material, and we observed a surprisingly rich impurity-induced phenomenology. We have carefully studied the origin of these "anomalous magnetization transitions" also using iPEPS simulations, which clearly revealed their underlying physics. Some of these discussions may have appeared confusing in our previous version of manuscript, and we have made extensive changes to improve our presentation in the revised manuscript. We hope the Reviewer finds the origin of the anomalies clear now in our revised manuscript.

Also, as the Reviewer pointed out, inducing superconductivity with chemical doping is beyond the scope of our current manuscript. We have therefore made the following changes to better reflect the focus of our paper.

a) We have modified the abstract and introduction of the paper on pages 1 - 3, and modified the text related to the superconductivity to put the focus on the effects of impurities on the magnetization process of the chemically doped Shastry-Sutherland system, to reflect our main results more clearly. Due to the length of the revised text, we respectfully ask the Reviewer to refer to our revised manuscript for the detailed changes. Please note that the modified text is highlighted for easier tracking.

b) On page 17 of the "Discussion" section, we have modified and abbreviated the text about the link between our final results and superconductivity in this system, so that it now reads:

"Moreover, these spin-singlet impurity pairs are fairly stable (binding energy $\gtrsim g\mu_B H'_{C0} \sim 1$ meV), and it is interesting to speculate the formation of Cooper pairs if holes (or electrons) can be associated with the impurities without too much energy cost. Note that the impurity pairs that we observe are strongly localized, suggesting that Cooper pairs, if present, might also be strongly localized. It is interesting to note that in cuprates as well as the doped $SrCu_2(BO_3)_2$ studied in the Ref. 11, the impurities reside in between the copper-oxide planes, while in $SrCu_{2-x}Mg_x(BO_3)_2$, Mg^{2+} replaces Cu^{2+} in the CuO_4 plane. Further studies are required to explore the difference between the impurity configurations in the two cases."

c) We have also modified the summary of the paper on page 17, which now reads:

"In summary, our results present the Mg-doped $SrCu_2(BO_3)_2$ as a model system for studying the effects of non-magnetic impurities in a frustrated quantum magnet, where results from theoretical simulation and multiple experimental methods can be directly compared. We have provided a clear description of the magnetization process for a Shastry-Sutherland system in the presence of impurities, which has a profound effect on the formation of the crystals of bound states of triplets. The results also reveal for the first time a rich impurity-induced phenomenology at fields below the magnetization plateaus, suggesting that even for samples with a Mg-doping as low as 1% ~ 2.5%, a single-impurity description such as that discussed in Refs. 15,16 is not enough to capture the essential physics, and interactions between the impurities and triplets must be considered. Further studies are desired to better understand the impurity-induced emergent states, to search for other possible novel phases (Bragg glass, Bose glass) at high fields, and to pursue the grand prize of RVB superconductivity."

As reflected in our revised manuscript, we believe our detailed study on the impact of impurities on the Shastry-Sutherland frustrated quantum magnet, a rare example for which a direct comparison between experiment and theory can be achieved to reveal the underlying physics, is a significant result that can be utilized to understand the relevant phenomena in a broad range of systems, and thus should be of interest to the readership of *Nature Communications*.

Furthermore, it looks that all transitions at lower and higher fields are suppressed with the increase in the temperature that may come out that both kind of transitions/plateaus have the same origin?

We appreciate the Reviewer's important observation, and are thankful for pointing out the need to further illuminate the presentation of one of the most important results of our paper. We have

indeed carefully studied the origins of the H'_{C0}, H'_{C1}, H'_{C2} and H'_{C3} anomalies, and compared them with the plateaus at higher fields. Please note that in the revised manuscript, the fields at which these high field plateaus are detected for doped samples are now called pseudo-1/n (n=2, 3, 8 ,…) plateaus. Please find the reason behind this terminology by referring to Reviewer 3 and our corresponding response, where we were advised to make such modification for clarity.

By careful examination of the temperature dependence of the observed features at both high and low magnetic field regions, we have found that the melting temperature for the H'_{C0}, H'_{C1}, H'_{C2} and H'_{C3} anomalies is slightly lower than those of the (pseudo-1/n) plateaus emerging at higher magnetic fields. For instance, in case of the x = 0.05 sample, the low field anomalies generally disappear at or below T ~ 2 K (Figs. 3d inset, S5d, and S6a), in contrast to T ~ 3K - 4 K, where the (pseudo-1/n) plateaus melt (Figs. S2d and S5d), suggesting their different origins.

To address the Reviewer's comment, we have added the following sentences at the bottom of page 9:

"These anomalies are found to be suppressed with increasing temperature (see Figs. 3d inset, S5 and S6). In the x = 0.05 samples, for example, they disappear at T ~ 2 K, before the pseudo-1/n plateaus melt at 3 K ~ 4 K (see Figs. S2d and S5d). The different energy scales suggest that the underlying spin structures of these anomalies are different from the superstructures of bound states of triplets associated with the pseudo-1/n plateaus. This is confirmed by our simulation results as discussed below."

Moreover, the signatures corresponding to the different origins of the low-field H'_{C0}, H'_{C1}, H'_{C2} and H'_{C3} anomalies and the high-field (pseudo-1/n) plateaus are also revealed in several other observations: First, the doping dependence shows contrary properties: while the (pseudo-1/n) plateaus are suppressed by doping (Figs. 1, 2, and 3), the low-field H'_{C0}, H'_{C1}, H'_{C2} and H'_{C3} anomalies are enhanced with doping (Fig. 3). Second, they show different lattice coupling characteristics: the magnetostriction measurements collected for the doped samples clearly show evidence for the (pseudo-1/n) plateaus (Fig. 1), while the absence of the H'_{C0}, H'_{C1}, H'_{C2} and H'_{C3} anomalies is only found within experimental resolution (Fig. 4). Third, the anomalies at H'_{C0}, H'_{C1} occur before the spin gap is closed, where the triplets underlying the (pseudo-1/n) plateaus are still absent, and therefore they should exhibit different origin. Forth, our iPEPS simulations imply the origins of the low-field H'_{C0}, H'_{C1}, H'_{C2} and H'_{C3} anomalies to be different than those proposed for (pseudo-1/n) plateaus. Considering that these simulation results are in good agreement with our experimental results, the prospect that such scenario is more likely are increased.

We have made the following changes to further address the Reviewer's comment:

a) On the top of page 9 of the revised manuscript, we have modified a sentence, and it now reads:

"Their doping dependence, however, are exactly the opposite: the H'_{C1}, H'_{C2}, and H'_{C3} anomalies are enhanced with higher doping concentration, while the pseudo-1/8 plateau is suppressed. This completely different behavior on doping suggests that these anomalies have origins that are different from the pseudo-1/n plateaus."

b) On page 10, we have modified related text, which now reads:

"This is unlike the strong lattice coupling observed for magnetization plateaus corresponding to the crystallization of bound states of triplets, again indicating their different origins. This interpretation is further strengthened by the fact that the H'_{C1} *and* H'_{C0} *anomalies appear at fields comparable with, or below, the gap closing fields at which bound states of triplets are absent and cannot play any role in their formation."*

Minor points:
1) dM/dH is also need for $x = 0$ for better comparison.

We thank the Reviewer for pointing out again the importance in making comparison between the undoped and doped samples. As we discussed above, such a comparison can be made from our TDO data, where df/dH ($\propto dM^2/d^2H$) provides the same information with higher resolution. Accordingly, the TDO results (Fig. 3c) clearly show the absence of the H'_{C0}, H'_{C1}, H'_{C2} and H'_{C3} anomalies for $x = 0$, whereas these results demonstrate the enhancement of these anomalies when the doping concentration x is increased.

2) H||a means in-plane field?

The Reviewer is correct that $H \parallel a$ corresponds to the in-plane field, and we thank the Reviewer for his/her comment. To address any confusion, we have modified the first sentence on page 3, which now reads:

"The parent compound of $SrCu_2(BO_3)_2$ *consists of two-dimensional layers of* Cu^{2+}*(S = 1/2) orthogonal dimers arranged on a square lattice (Fig. 1a inset), which form the ab-plane of its tetragonal unit cell."*

3) I understand that authors presented dH/dM data in figure 2 to elaborate the transitions. But, dH/dM is mathematically not correct since you are measure M as a function of H?

We appreciate the Reviewer's comment, and it is correct that we actually measure M as a function of H. In Fig. 2, by dH/dM, we were referring to the inverse susceptibility $(dM/dH)^{-1}$. As the Reviewer pointed out, we performed this analysis following the method adopted from S. E. Sebastian *et al.*, *Proc. Natl. Acad. Sci. USA* **106**, 20157-20160 (2008), since it allows more accurate determination of the transition field. However, we agree that the used terminology does not represent the actual measured physical property accurately and we thank the Reviewer for pointing this out. In response to the Reviewer's comment, we have replaced "dH/dM" with "$(dM/dH)^{-1}$" throughout the paper.

In summary, this is an intriguing manuscript but there are several issues connected to origin of the anomalous magnetization jumps. Effect of doping concentration and temperature is also not very clear. More detailed interpretation and discussions may be needed.

We thank the Reviewer for recognizing the significance of our results, and are grateful to her/him for pointing out the areas of improvement in our presentation. Inspired by the Reviewer's valuable comments, we have significantly revised the manuscript and included additional detailed interpretation and discussions, which we believe considerably improve our presentation. In our revised manuscript we have added discussions illuminating the importance of our findings: explaining the discovery of such a rich impurity-induced phenomenology, as well as demonstrating their underlying physical origin with compelling evidence from complementary experimental techniques and theoretical simulation results. We hope that the Reviewer finds our implemented discussions illuminating and agrees with us that the added detailed interpretations now provide a more coherent and clear picture of the impurity effects in chemically doped magnetically frustrated Shastry-Sutherland system, making our revised manuscript suitable for publication.

Reviewer #3

In this paper, the authors report unexpected anomalies in the magnetization curve of doped SrCu2(BO3)2, also providing numerical calculations based upon the Heisenberg model on the Shastry-Sutherland lattice.

The paper is interesting but I have a few questions:

We thank the Reviewer for a careful reading of our manuscript and for appreciating the significance of our findings. The Reviewer has raised several very interesting and important questions, which have prompted us to improve our manuscript significantly.

1) First of all, I do not understand the definition of M_{ref}=3*M_{1/3}. The authors say that "M_{1/3} refers to the magnetization at the 1/3 plateau", which should be 1/3 by definition, for all dopings. So M_{ref}=1 always.

We are grateful to the Reviewer for pointing out the confusion and the need for clarification. The definition of the reference magnetization M_{ref} is indeed a very important subject in the context of the doped system. As we demonstrate below and in the revised manuscript, the introduction of M_{ref}, in addition to the true saturation magnetization M_{sat}, is necessary to achieve a clear description for the magnetization process for the *doped* samples. Please note that the Mg^{2+} is substituted for Cu^{2+} in doped systems. Consequently, in case of the doped samples, because of the reduced number of magnetic Cu^{2+} moments, the true saturation magnetization M_{sat} should actually be less than 1 when compared to the undoped system. However, as we show below, there is still important difference between M_{ref} and M_{sat}, which has prompt us to make extensive modifications in our manuscript to clarify this point.

We have realized that much of the confusion arises when the terminology "1/n-plateau" (where n=3, 4, 8, …) is used for both undoped sample (with $x = 0$) and for the doped samples (with $x>0$).

We have, therefore, reserved the term "1/n-plateau" to refer only to the undoped sample, while we use the term "pseudo-1/n plateau" for the doped samples. As the Reviewer correctly pointed out, the "pseudo-1/n plateau" terms in the doped samples, do not actually correspond to the 1/3, 1/4, and 1/8 of the explicit saturation magnetization M_{sat} which can only be obtained in the undoped sample. Therefore, it is more accurate to use the term "pseudo-1/n plateau" in case of doped systems. Accordingly, in the doped system, the M values at "pseudo-1/n plateaus" are found to be fractions (1/3, 1/4, 1/8) of a magnetization value slightly less than the saturation magnetization value M_{sat}, which we call reference magnetization M_{ref}. In our paper, the values for M_{ref} for each doping concentration are extracted from the magnetization values at the "pseudo-1/3" plateaus. For the undoped sample, M_{ref} corresponds to the magnetization value M_{sat} and it can similarly be defined using the 1/3 plateau. Therefore, one can consider M_{ref} as a more general term than M_{sat}. It is noteworthy that in case of the doped samples, the difference between M_{ref} and M_{sat} is attributed to the the softening of the crystals of bound states in the presence of impurities.

To address the Reviewer's comment, we have expanded the corresponding text and included more detailed discussions in our revised manuscript. The changes are summarized below.

a) For all the *doped* samples the "1/8", "1/4", and "1/3" plateaus are renamed as "pseudo-1/8", "pseudo-1/4", and "pseudo-1/3" plateaus. This change is made throughout the revised manuscript. We note that for the *undoped* sample, the meaning of "1/8", "1/4", and "1/3" plateaus are reserved, consistent with the convention used in the literature.

b) In the section of "Magnetization plateaus", a significant part of the text has been rewritten and expanded. One relevant paragraph has also been moved to this section from the "Discussion" section and modified. The revised text includes four paragraphs from page 5 to the beginning of page 8 in the revised manuscript:

"In doped $SrCu_2(BO_3)_2$, Fig. 1a clearly shows that the magnetization is suppressed with increasing doping, suggesting a suppressed density of triplets in the presence of impurities.
…
It is interesting to speculate how this process takes place, and extremely high magnetic fields are required for such a study."

Due to the length of the revised text, we respectfully ask the Reviewer to refer to our revised manuscript for detailed changes. Please note that the modifications to the text are highlighted for easier tracking.

c) In the "Discussion" section, we have added one new paragraph to discuss the implications of the softening of the superstructures of bound states in the doped samples, revealed by the difference between M_{ref} and M_{sat}. The added interpretation is on pages 15 - 16, and reads:

"At high magnetic fields, the presence of impurities is found to disrupt the formation of superstructure of bound states of triplets and breaks it into small patches. In the x=0.05 sample, these patches of superstructures account for ~ 85% of the total Cu^{2+} moments, as shown earlier. This significant softening of the superstructures is the reason for the smearing

of the pseud-1/n plateaus in the doped sample. It is expected that the pseudo-1/n plateaus would eventually be totally suppressed as the superstructures are further disrupted with increasing doping. Indeed, the fate of symmetry-breaking phases in the presence of impurities is itself of great current interest[52]*. For the crystals of bound states formed at the magnetization plateaus of* $SrCu_2(BO_3)_2$ *(Ref. 22, 23), the non-magnetic impurities generically break the symmetries of their order parameters and play the role of random field disorder*[52]*. Therefore, it is interesting to speculate if there exists impurity-induced Bragg glass phase analogous to that in a type II superconductor*[53]*. It is also intriguing for future studies to search for other novel phases such as Bose glass, which has been found near Bose-Einstein condensate phase in other doped quantum magnet*[54]*."*

2) In the inset of Fig.1a, the curve for x=0.02 is above the one for x=0.03 for H<2. Is it clear the reason for this behavior?

We thank the Reviewer for the careful observation and for bringing this issue to our attention. Please note that the data presented in previous version of our manuscript shown in Fig 1 for x = 0.02 and 0.05 cases is obtained from the 30T shots, and the data for x = 0.03 sample is obtained from the 60T shot. Therefore, the inconsistent behavior of the x = 0.03 sample at low fields (below 2T) is likely due to the fact that these data sets were obtained from different field shots. We note that the accuracy of a high-field shots using the pulsed field technique is only limited at very low field region (below 2T), and the limit is only quantitative rather than qualitative. Therefore, this does not have any affect our conclusions which result from the features emerging at significantly higher fields.

In response to the Reviewer's comment, we have made the following changes for Fig. 1a.

a) Since the range of interest is at higher magnetic fields, we changed the x-axis range so that it starts from 2T instead of zero field to avoid any confusion, as the goal is to bring the focus of the readers to the field region above ~ 6T, instead of the field region below 2T, which is not the focus of this paper.

b) We present the data from 60 T shots for all three doping concentrations for better consistency, so that they can be compared more easily. It is now clear from the revised figure that the magnetization in the low field region does increase with doping, as expected.

3) Similarly, I am a bit surprised about the non-monotonic behavir of the magnetization curve of Fig.2, especially when considering the undoped case with x=0.

We appreciate the Reviewer's attention and careful observation. We note that considering the full field range, the *overall trend* of the results demonstrates that the magnetization curve for each doping is a monotonic function of field, i.e. dM/dH is always positive, and in order to make this clear, we have added labels to the y-axis of Fig. 3b.

With regards to the x=0 data, however, please note that the x = 0 trace is reproduced from Ref. 24 and that what is presented in Fig. 2 is in fact not magnetization, but *normalized* magnetization. By doing so, our intention has been to demonstrate that when normalized with respect to M_{ref}, the

traces for the doped samples can be collapsed on top of that for the undoped sample, with small difference between traces which may be limited by the finite accuracy of the data. We also note that Fig. 2 also shows that the pseudo-1/n plateaus are increasingly rounded for higher doping concentrations, and this observation is indeed consistent with our interpretation that the crystal of bound states is softened by impurities.

4) In Fig.3a, for H<25, the curves for x=0.03 and 0.05 almost coincide, while the one for x=0.02 is much below, suggesting a saturation when x>0.03. Do the authors have an explanation for that? It would be also nice to investigate this issue by using iPEPS.

We thank the Reviewer for the constructive suggestion. We note that it is very challenging to extract a qualitative doping dependence based on just three doping levels. We agree with the Reviewer that it would be interesting to study the doping dependence also with iPEPS. However, please nota that such a study would be suitable as an independent theoretical endeavor due to its time consuming and computationally demanding nature. Here we have been trying to keep the main focus on the striking discovery of a rich impurity-related phenomenology using different experimental techniques as well as on the suggestive underlying physics using the presented theoretical interpretations. We hope that these results inspire the community to conduct further theoretical and experimental investigations, and we do agree with the Reviewer that future studies representing more doping concentrations are certainly desired and beneficial.

5) The setup of iPEPS is such that the disorder is not a real disorder (the 12x12 unit cell is periodically repeated), I am wondering how much the estimations of H_{c2} and H_{c3} depend on the disorder configuration that has been chosen. I would be happy to have an errorbar on the determination of these critical fields: for example the magnetization curve obtained within iPEPS is not smooth (Fig.5a), and determining the change of slope can be affected by the fitting procedure: to be honest, I can even trace a single line (i.e., without visible anomaly) fitting the iPEPS data.

We appreciate the practical remark pointed out by the Reviewer. We agree that the disorder used here is not a "real disorder" of an infinite system, and that certain details of the magnetization curve may depend on the particular disorder realization used here. However, we point out that a 12x12 unit cell (288 spins) is considered to be remarkably large – for example significantly beyond what can be achieved by other techniques in comparison – providing already a notable accuracy of the critical fields in the limit of infinite unit cells (real disorder). This is supported by the fact that the critical field we find for H'_{C2} (0.428J) in this cell is very close to the value of an isolated localized bound state (0.427J), which also corresponds to the value of an infinite system with "true" disorder (since every infinite system will contain locations in the lattice where quasi-isolated localized bound state can be formed). Similarly, we do not expect a significant (i.e. beyond a few percent) finite-unit-cell deviation in H'_{C3}.

We would like to emphasize here that the goal of the iPEPS simulations was to obtain a qualitative/semi-quantitative understanding of the anomalies observed in experimental results, in particular please note that determining the critical fields with an extremely high accuracy is out of the scope of this study.

More importantly regarding H'_{C3}, we would like to stress that an abrupt change of slope is not required, since a rapid change of slope can sufficiently explain the results. This can be interpreted considering that in the magnetization measurements, such a rapid change manifests as a "bump" in dM/dH. In the TDO measurements, the data can be related to the second derivative of $M(H)$, and a rapid change of the slope already yields an observable peak in the data.

The standard approach that is used in case of finite-size data (in our case finite unit-cell data) is to examine the centers of each plateaus, usually by drawing the curve which connects them.

To address the comment pointed out by the Reviewer, in the revised manuscript, we mark the centers of each plateaus by the black dots in Fig. 5a. We believe this attempt should better feature the change of slope occurring near H'_{C3}. This should be particularly helpful, since it is not possible to satisfactorily fit the black dots (centers of plateaus) with a single straight line – instead, it is now clear that the linear fits below and above H'_{C3} can describe the data more accurately. In our revised manuscript, H'_{C3} is defined as the point where the two line-fits meet.

In addition, in response to the Reviewer's constructive suggestion, we have also included error bars for H'_{C2} and H'_{C3}, which reflect the resolution in h/J in the simulation results: on page 12 we have "$H'_{C2} = (0.428 \pm 0.001)J$ (~ 21.8 ± 0.1 T)", and on page 13 we have "$H'_{C3} = (0.452 \pm 0.004)J$ (~ 23.1 ± 0.2 T)".

Finally, we note that what is more important than the actual value of the slopes in this study, is the underlying qualitative change of how the lattice gets populated - i.e. by only bound states below H'_{C3} and by bound states and additional triplets for H higher than H'_{C3}, which naturally leads to a qualitative change in the magnetization curve as a function of H at H'_{C3}. We stress this point more clearly in the iPEPS section (second paragraph on page 13) of the revised manuscript.

Moreover, it is written that "this suggests that the anomaly observed at H_{c3} ~ 25T in experiments is due to a change of slope in the magnetization curve". I find this claim quite strange, since, by looking at the experimental data, it should be easy to see if there is a change in the slope or not. May the authors be more precise on that?

We thank the Reviewer for the useful comment. What we meant is that H'_{C3} corresponds to a "change of slope", not a "plateau". To clarify this issue, we have modified the text accordingly and page 13 of the revised manuscript now reads:

"*Thus, this suggests that the anomaly observed at H'_{C3} ~ 25 T in experiments is due to a rapid change of slope…, and it is not a magnetization plateau.*"

We note that one could indeed observe the "change of slope" in Fig. 3b, where dM/dH shows a "bump" for the doped samples, which is more clearly seen in the more sensitive TDO data presented in Fig. 3c which is proportional to the second derivative of the magnetization. Also, please note that the data may appear to be rounded because of finite temperature effects.

6) In addition, it is claimed that the value of H_{c2} obtained by iPEPS with disorder is not very different from the one without disorder. However, for x=0, the experimental data do not show a significant singularity at H_{c2}. Therefore, I am not sure if the way to determine H_{c2} within iPEPS is correct or not.

We appreciate the Reviewer's important observation. Please allow us to clarify this point: without impurities the bound states are actually *delocalized* in the dilute limit of excitations and we expect a smooth increase of the magnetization, i.e. without a jump/anomaly at H'_{C2}. The statement we made in the paper is that H'_{C2} is close to the field which is *necessary* to create a localized bound state (without impurities) which was computed in Ref. 23. However, for the undoped sample, the excitation energy to create localized bound states is higher than the one of a delocalized bound state. Therefore, only *delocalized* bound states appear in the undoped samples. For the doped samples, however, localized bound states are the preferred ground states because of the presence of impurities.

In response to the Reviewer's comment, we have made the following changes in the revised manuscript.

a) On page 13 of the revised manuscript, the text of the first paragraph is modified, and it now reads:

"It is interesting to point out that H'_{C2} is close to the energy scale necessary to create a localized bound state in systems without impurities, 0.427J (Ref. 23).
...
Therefore, in undoped systems, bound states are delocalized in the dilute limit of excitations[42], and a smooth increase of the magnetization, i.e. without a jump (anomaly) at H'_{C2}, is expected."

b) On page 16, the modified text reads:

"In the undoped system, the energy cost for creating localized bound states is higher than that for the delocalized ones.
...
Therefore, only in the presence of impurities, the H'_{C2} and H'_{C3} anomalies are realized."

From iPEPS, H_{c2} is associated to the existence of a localized bound state: I am wondering if this statement can be confirmed within the experimental setup.

We thank the Reviewer for the very interesting suggestion. As we explained above (page 11 of this reply), our iPEPS simulation results, obtained for a very large unit cell size, provide an accurate account for the origin of H'_{C2} - which is confirmed by the remarkable agreement between these results and our experimental data obtained for H'_{C2}. Such a good agreement provides a credible support for our interpretations. Having said that, we agree with the Reviewer that it is desirable to perform other experiments to confirm the reported phenomena, however, we note that such a change in the magnetization process is best studied using probes sensitive to the magnetization, which is exactly what we conducted using four complementary experimental techniques. Thermal transport maybe another technique that can be used to study the localized vs

delocalized nature of the bound states. Therefore, we agree with the Reviewer that it would be an interesting experiment for future study but it is beyond the scope of our current study, and we hope these results encourage the community to continue these investigations in future using relevant techniques.

Minor points:
1) Why is the acronym of the Shastry-Sutherland model SSL and not just SS?

We appreciate the Reviewer's suggestion on the acronym of the Shastry-Sutherland model. We have taken the Reviewer's advice and changed it into "SS" throughout the paper.

2) I think that in the introductory part too much emphasis is put on the possibility to have superconductivity. This aspect is instead not addressed in the paper. I suggest to smooth these statements.

We thank the Reviewer for her/his constructive suggestion. We agree with the Reviewer that some of the discussions on superconductivity are not essential to the message of the paper and maybe distracting. We have, therefore, made extensive changes to the abstract, introduction, and discussion in our revised manuscript. Our detailed reply and summary of the changes are listed in our response to the similar comment by Reviewer 2 - please see pages 5 - 7 of this reply.

In summary, I think that the paper can be published, but only after the authors will address the points raised above.

We are grateful to the Reviewer again for the careful reading of our manuscript, and we are pleased that she/he has recommended our paper for publication once we address his/her comments. As demonstrated in this reply and in our revised manuscript, we have made extensive changes to make sure we fully address all of the Reviewer's concerns. Therefore, we hope that the Reviewer finds our response satisfactory and our revised manuscript suitable for publication.

REVIEWERS' COMMENTS:

Reviewer #1 (Remarks to the Author):

The authors have properly reply the concerns raised by the referees and revised the manuscript accordingly. Now I recommend to accept this paper for publication in Nature Communications.

Reviewer #2 (Remarks to the Author):

The authors have provided detailed replies to all of my queries and have revised the paper accordingly. I am satisfied with clear replies, illuminating discussions and necessary revisions from authors. The revised manuscript will be rather effective now. Therefore, I recommend the paper for publication in the Nature Communications.

Reviewer #3 (Remarks to the Author):

I have seen the revised version of the manuscript. Indeed, the authors made significant modifications to answer my previous questions. In my opinion, the present version of the paper may be published in Nature Communications.

Reply to the comments by the Reviewers

Reviewer #1 (Remarks to the Author):

The authors have properly reply the concerns raised by the referees and revised the manuscript accordingly. Now I recommend to accept this paper for publication in Nature Communications.

Reviewer #2 (Remarks to the Author):

The authors have provided detailed replies to all of my queries and have revised the paper accordingly. I am satisfied with clear replies, illuminating discussions and necessary revisions from authors. The revised manuscript will be rather effective now. Therefore, I recommend the paper for publication in the Nature Communications.

Reviewer #3 (Remarks to the Author):

I have seen the revised version of the manuscript. Indeed, the authors made significant modifications to answer my previous questions. In my opinion, the present version of the paper may be published in Nature Communications.

We are pleased that three Reviewers found our responses and revisions satisfactory. We are grateful to the Reviewers for their valuable time reviewing our manuscript, for their comments which has improved our manuscript significantly, and for recommending the publication of our work in Nature Communications.
